# The Kasei Valles, Mars: a unified record of episodic channel flows and ancient ocean levels

**DOI:** 10.1038/s41598-020-75080-y

**Published:** 2020-10-29

**Authors:** Sergio Duran, Tom J. Coulthard

**Affiliations:** Energy and Environment Institute, University of Hull, Hull, UK

**Keywords:** Geomorphology, Hydrology, Inner planets

## Abstract

There is widespread evidence across Mars of past flows in major channel systems as well as more than one palaeo ocean level. However, evidence for the timing of channel flows and ocean levels is based on geographically diverse sources with a limited number of dates, making reconstructions of palaeo flows and ocean levels patchy. Here, based on high-resolution topography, image analysis and crater statistics, we have dated 35 different surfaces in Kasei Valles, that are predominantly found within erosional units enabling us to reconstruct a fascinating timeline of episodic flooding events (ranging from 3.7 to 3.6 Ga to ca. 2.0 Ga) interacting with changing ocean/base levels. The temporal correlation of the different surfaces indicates five periods of channel flows driving the evolution of Kasei Valles, in conjunction with the development of (at least) two ocean levels. Furthermore, our results imply that such ocean rose in elevation (ca. 1000 m) between ca. 3.6 Ga and 3.2 Ga and soon afterwards disappeared, thereby indicating a complex ancient Martian hydrosphere capable of supporting a vast ocean, with an active hydrological cycle stretching into the Amazonian.

## Introduction

The surface morphology of Mars suggests that water—or other liquid flows—have played an important role in shaping the planet^1–5^. Huge channels, valleys and canyons—with widths of tens of kilometers and a thousands of kilometers in length—give evidence of large quantities of liquid water that once flowed over the Martian surface^3,5,6^. In addition, the flow termination point of many of the large Martian channels is consistent with the existence of an ancient Northern ocean^7^. Accordingly, planet-wide shorelines, tsunami deposits and delta elevations on the southern edge of the Northern plains have been interpreted as evidence of a past Northern ocean^2,7–12^. Recent research added further evidence identifying knickpoints in multiple Martian channels that occur at two common elevations, suggesting two former ocean levels broadly in agreement with the elevation from the delta, tsunami and shoreline deposits^13^. If early Mars had a global hydrosphere cycling water into a Northern ocean, reconstructing the timing of flows in Martian channels and dating past ocean levels could provide critical insights into planet-wide hydrological trends and changes of ocean levels during the early geological history of Mars. Whilst we already have some insights into when channels were flowing, they tend to only show single times when individual channels were flowing^3,5,6^ painting an incomplete picture of past flow records.

The inventory of fluvial landforms on Mars includes multiple features previously observed and examined on Earth, such as meander belts^14,15^, paired and unpaired river terraces^15,16^, incised inner gorges^6,17,18^ and/or fluvial knickpoints^16,19^. On Earth, river terraces and channel exposures can be dated and fluvial landforms and channel long profiles mapped to allow the reconstruction of a fluvial history^15^. For example, mapping series of terrace surfaces can show past long profiles and if dated when that profile changed via incision. In addition, dating abandoned fluvial surfaces or channels can provide insights into when that system were last flowing at that location and identifying common knickpoints can be used as a record of past base levels. Therefore, if we can reconstruct the history of how Martian fluvial geomorphology has evolved, this can be a powerful tool to understand past environmental conditions on Mars.

Here, we combine new image and topographic data to map river channels and fluvial landforms in the Kasei Valles system, the largest outflow channel on Mars^5^. Furthermore, we use these high resolution topographic data with crater size-frequency distribution statistics to date when channel surfaces and features were created and last re-worked. This has allowed us to reconstruct a three dimensional history of the development of the Kasei Valles from the early Hesperian (ca. 3.6 Ga) to early Amazonian (ca. 2.0 Ga)—ages herein based on Hartmann chronology^20^. This reveals a fascinating timeline of the long-term evolution of Kasei Valles, showing five episodes of channel incision/activity, in conjunction with a changing (rising) global ocean/base level during the Hesperian period.

## Methods

**Figure 1:**
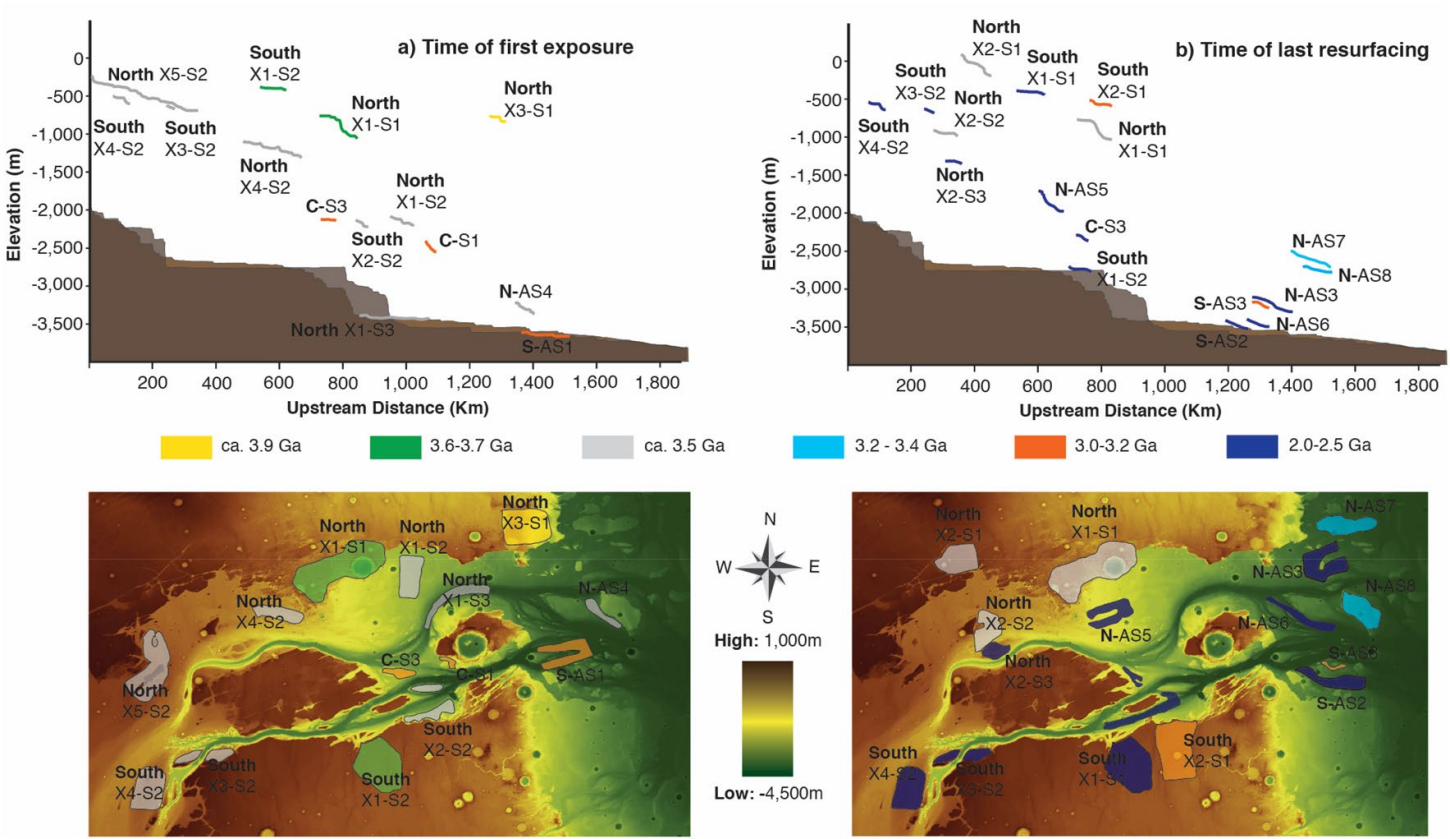
Representation of the evolution of the longitudinal profile of Kasei Valles. On the left are two panels where the different colors represent the first exposure of surfaces within Kasei Valles. On the right, the panels with different colors represent the date of last resurfacing/abandonment of surfaces within Kasei Valles. At the top are the longitudinal representation of these surfaces. At bottom are the spatial distribution of these surfaces. The coding indicates the cross section number (X), the surface within the cross section (S) and whether it is in the North or South tributary. Alternatively, the coding indicates when the surface correspond to an abandoned channel (AS) or surface formed by the river capture (C). The base map for both panels (a, b) is a color-coded shaded-relief MOLA DEM (460 m/pixel). Credit: MOLA Science Team, MSS, JPL, NASA. We produced these maps in this figure using Esri’s ArcGIS 10.6 software (https://www.esri.com/software/arcgis) and the mosaic using Adobe’s Illustrator CS6 software (https://www.ado-be.com/es/products/illustrator.html).

The Kasei Valles are the largest outflow channels on Mars, with a channeled area extending 2000 km in length and a channel width of up to 150 km^5^ (Fig. 1 & Supp. Figure 2). The channels exhibit high width/depth ratios, low sinuosity (except at the channel mouth, where meandering reaches are observed), paired fluvial terraces, fluvial benches and several major knickpoints^6,21^. Previous mapping studies proposed a model for the erosion/evolution of Kasei Valles involving four periods of water activity and (at least) four episodes of volcanic activity^22–24^.

To reconstruct the formation and evolution of Kasei Valles, we used the Mars HRSC MOLA Blended global 200 m resolution Digital Elevation Model (DEM). Using Arc-GIS we extracted the channel long profiles with a series of cross sections to identify surfaces adjacent to the channels (Supp. Figure 1). Subsequently, we extended our mapping between cross sections where there were common flat surfaces and we identified any additional/abandoned channels for further analysis. To date these surfaces and channels, we imported Mars Reconnaissance Orbiter high-resolution Context Camera (CTX) imagery into Arc-GIS and analyzed the crater population within each surface. The analysis of crater size-frequency distribution (CSFD) requires a surface large enough to contain a representative number of craters and was therefore limited by the surface homogeneity and area. On this basis, we defined the counting locality (ideally approaching to 10,000 km^2^, but setting a minimum threshold of 1000 km^2^ as this count size introduces low uncertainties^25^) and estimated the crater population using the Arc-GIS extension *‘CraterTools’*^26^. We mapped every identifiable crater with a diameter ≥ 200 m and exported the data into the software *‘CraterStats’*, that analyses the spatial randomness and clustering of the crater population^26^. Subsequently, we determined the absolute model ages of the crater population analyzed by fitting parts of the CSFD that (1) fit well to the production functions and (2) are comprised by a minimum of four bins of randomly distributed diameters. In cases where resurfacing processes might have affected the crater samples, two or more model ages per surface could be obtained (Supp. Figure 3 & 4). We finally assessed whether we needed to apply a resurfacing correction to the model age determined (Supp. Figure 3).

Subsequently, we classified each absolute model age as either *date of first exposure/formation* or *date of last resurfacing.* We defined the former as being the period of time elapsed since craters started collecting on the surface, whereas the latter as being the time elapsed since a minor modification of the crater population occurred (Supp. Figure 4). For counting areas < 5,00 km^2^, we visually inspected the surface using CTX and HiRISE imagery to determine if the observations correspond to the most surficial unit (last resurfacing period) or were an underlying unit partly buried by younger one/s.

## Results and interpretations

35 surfaces and channels across Kasei Valles were identified and dated through counting more than 50,000 craters, as shown in the Supp. Figure 2. The calculated surface and channel ages clustered into 5 time periods from 3.7–3.6 Ga to 2.0 Ga (Supp. Figure 19). These dated surfaces were plotted in plan view and longitudinal profile in Fig. 1 and colored according to six time periods, where the yellow color represent Noachian rock-age surfaces predating the development of Kasei Valles^22,27^. These data reveal a complex and episodic history of flooding events driving the co-evolution of the branches of Kasei Valles system, including an example of river capture or piracy^28^ (Supp. Figure 10).

The dates of first exposure for the surfaces within Kasei Valles (Fig. 1a) show a systematic chronology of channel evolution with the oldest surfaces being the highest (3.7–3.6 Ga) and the main path of the Kasei North and South tributaries being carved around 3.5 Ga. The long profiles for first exposures at this 3.5 Ga flooding period align along a common slope for both North and South tributaries (Fig. 1a; grey surfaces), thereby suggesting that extensive and episodic flows carved and shaped the landscape there. Older dated surfaces (3.7–3.6 Ga) similarly align along common slopes (Fig. 1a, green surfaces), with the age of the long profiles becoming younger downwards. Importantly, this chronology indicates that the valley was cut down relatively quickly by two early flooding episodes (3.7–3.6 Ga and ca. 3.5 Ga respectively; green and grey surfaces in Fig. 1a) and subsequently shaped over several hundreds of million years by periods of water activity (Fig. 1a,b) consistent with previous investigations^22,23^. Further dated surfaces reveal that the Northern tributary was captured by the South tributary around 3.2—3.0 Ga (C-S3 & C-S1, Fig. 1a and Supp. Figure 2). This is evidenced by the 3.5-Ga surfaces North X1-S3 and N-AS4 (Fig. 1a), being downstream of the capture zone at low elevations—where craters were preserved by the bulk of the flow switching to the Southern tributary. Furthermore, in the Southern tributary below the capture zone there is evidence of increased later discharges with surfaces being reworked at low elevations (S-AS1, Fig. 1a), and channel incision leading to abandoned adjacent surfaces at higher elevations (S-AS3, Fig. 1b). In addition, these low-elevated surfaces (S-AS1 & S-AS3) indicate that the base-level for this period of channel activity (3.2–3.0 Ga) was low (< − 3500 m), enabling the channels to incise down to greater depths than previously.

**Figure 2:**
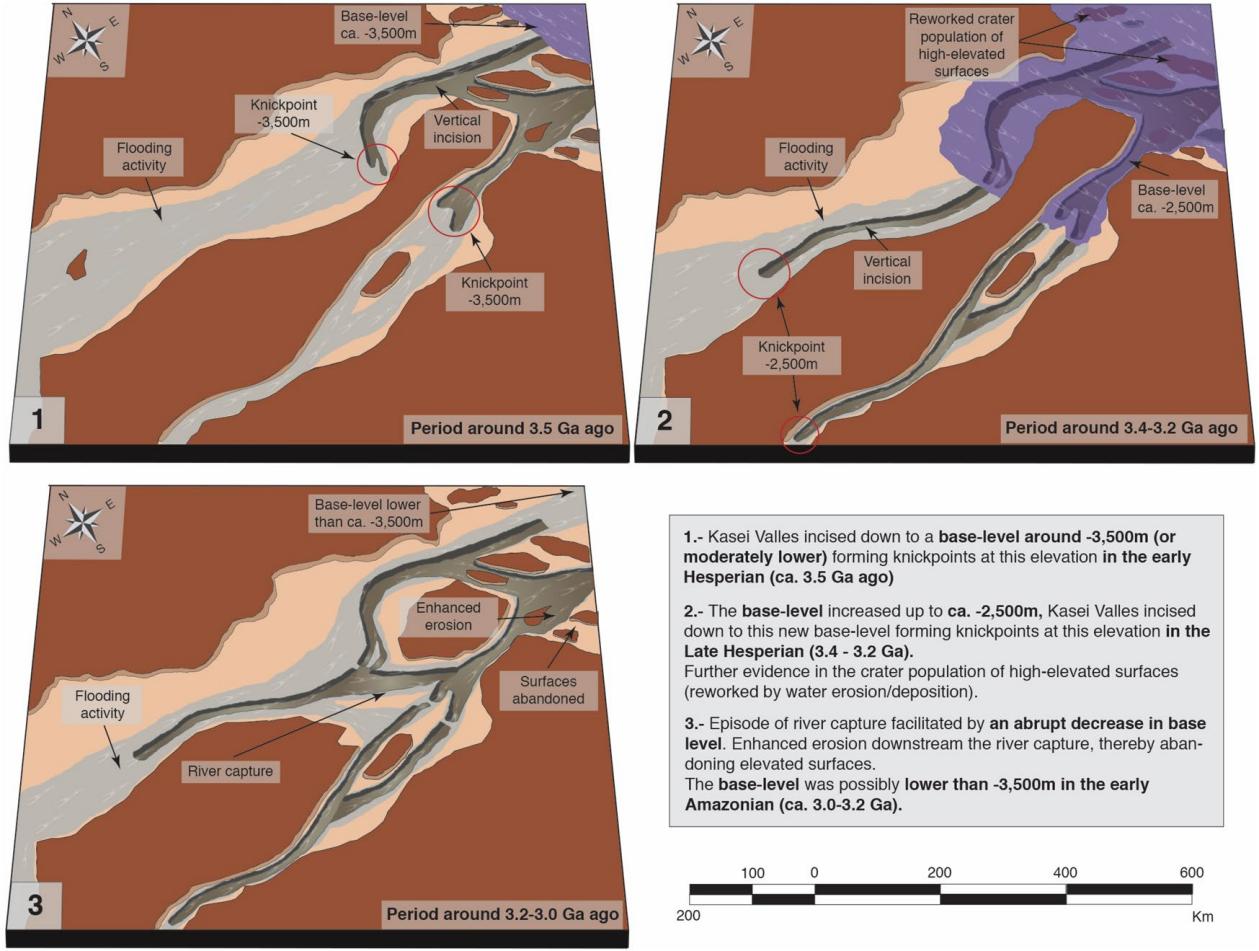
Schematic representation of the evolution of Kasei Valles’ base-level over time. The figure explains, in 3 steps, the evidence for the evolution of Kasei Valles’ base-level. Each step is represented by a block and each corresponds to a different time period, Blocks 1, 2 & 3 correspond to the periods ca. 3.5 Ga, 3.4–3.2 Ga and 3.2–3.0 respectively. The middle part and mouth of Kasei Valles is sketched within each block. The location of geomorphological features (such as knickpoints) and dimension of geomorphic attributes (such as channel width or depth) are illustrative rather than exact. Thus, the scale bar aims to give a sense of the dimension, rather than an exact magnitude. We produced this figure using Adobe’s Illustrator CS6 software (https://www.adobe.com/es/products/illustrator.html).

Whilst the bulk of Kasei Valles development is revealed by the first exposure dates, the date of last resurfacing (Fig. 1b) provides information about when the channels were last flowing. These data indicate that episodic channel activity in Kasei Valles was ongoing until around 2.0 Ga (Early Amazonian), which has been also proposed in previous investigations^23^. However, there are two important caveats considering the last resurfacing data: Firstly, the resurfacing might result from flows sufficient to smooth the surface, but not sufficient to carve major features—which, in turn, could indicate a weakening of this hydrological cycle over time. Secondly, other geological processes (e.g. aeolian activity or volcanism) could also be responsible for the resurfacing. However, (i) the correlation in time of last resurfacing between more than 1 surface, (ii) the relationship between the last resurfacing time and elevation (with the age of last resurfacing generally becoming younger downwards in elevation) and (iii) the similar range of crater diameters removed/obscured in surfaces affected by each last resurfacing episode reduces the likelihood that these events are a result of non-fluvial processes (e.g. aeolian activity or volcanism) (Fig. 1b).

**Figure 3:**
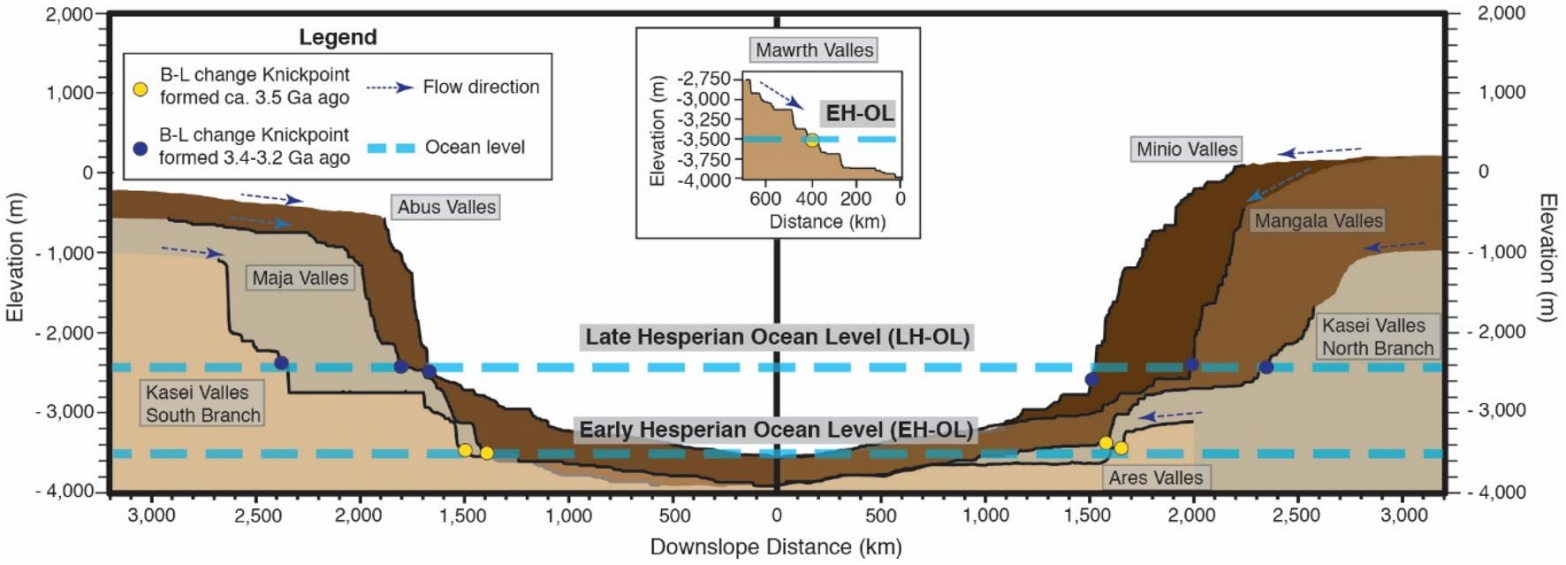
Representation of the longitudinal profiles of outflow channels exhibiting base-level change knickpoints and the location of these knickpoints. The graph is split into two parts: on the left, it shows the longitudinal profiles of Abus, Maja and the Southern branch of Kasei Valles. On the right, it displays the longitudinal profiles of Minio, Mangala, the Northern branch of Kasei Valles and Ares Valles. The area below each longitudinal profile has been given a different color to facilitate the visualization. A thick black line represents the actual longitudinal profile; the rest broadly represents the topography of the upstream terrain. A yellow circle indicates the location of base-level change knickpoints formed in the early Hesperian (ca. 3.5 Ga). A blue circle indicates the location of base-level change knickpoints formed in the late Hesperian (3.4–3.2 Ga). The two blue thick dashed lines illustrate the elevation of the ocean during the formation of these two group of knickpoints. We produced this figure using Adobe’s Illustrator CS6 software (https://www.adobe.com/es/products/illustrator.html).

The long profiles analyses clearly display that both—North and South—tributaries contain major knickpoints at ca. − 3500 m and − 2500 m. Importantly, major knickpoints at similar elevations are also found in other outflow channels such as Maja Valles, Mangala Valles and Ares Valles (Fig. 3). Traditionally, these theatre-shaped knickpoints have been attributed to episodes of flooding erosion over layers of different resistant rock^6,29^,. However, recent investigations indicate that their shared elevation and distribution across the planet are more consistent with global controls (i.e. different episodes of flood erosion incising down to global base/ocean levels)^13^. Thus, these knickpoints represent a record of past ocean levels—at ca. − 3500 m & − 2500 m—and, by using these ocean levels in our analysis, we can reconstruct in greater detail the evolution of Kasei Valles.

The formation and evolution of Kasei Valles appears to be marked by three distinct phases, as illustrated in Fig. 2. At around 3.7–3.5 Ga, two major flooding periods eroded the vast majority of the pre-flooded highlands (units Hr & Nplh according to references 22, 27), incising down to a base-level at ca. − 3500 m (or slightly lower), which coincides in time and elevation with both the Deuteronilus shoreline contact^2,10^ and other palaeo-shorelines previously mapped from tsunami deposits^8^. As a result, knickpoints formed and migrated hundreds of kilometers upstream in both North and South tributaries (Fig. 2.1). Subsequently, up to around 3.4–3.2 Ga the base-level increased in elevation, reaching an altitude of ca. − 2500 m. During this high-stand, subsequent channel flows eroded the long profile down to this base-level, thereby forming the knickpoints observed around this elevation (Fig. 2.2). Further evidence of surfaces from this period can be seen in the last resurfacing time of high-elevated surfaces at the mouth of Kasei Valles (N-AS7 & N-AS8, Fig. 1b & Supp. Figure 9), that could not have been inundated unless the base-level had risen. Existing studies suggest that this could have been a period of sustained fluvial activity in Kasei Valles rather than larger individual events^22,30^. This hypothesis is consistent with our observations and explains the lack of major features carved during this period, as well as the much smaller size of the knickpoints located at this zone (compared with knickpoints formed during the previous flooding periods). Importantly, this coincides in time with the late stage of fluvial incision that eroded valley networks^14^ and emplaced deltaic^1,14^ deposits mapping a palaeo-shoreline at around the same elevation^1,14^. Finally, the base level fell down abruptly around 3.2–3.0 Ga, leading to the river capture of the Northern tributary by headward incision from the Southern tributary (Fig. 2.3).

Our reconstruction of episodic flows working with a changing ocean level is supported by separate studies from other outflow channels and coastline deposits on Mars. Channels of the Ares, Maja and Mawrth Vallis all indicate a major pulse of activity around the Late Noachian—early Hesperian (ca. 3.5–3.7 Ga)^31–33^ (Table 1). The Ares, Maja and Mawrth Vallis also exhibit knickpoints around − 3500 m (Fig. 3) with shorelines that have been dated to ca. 3.6 Ga^2,8^ at − 3500 supporting our interpretation that channels were incising to a common Northern ocean level of − 3500 m during this period. Additional analyses on Ares Vallis further reinforce that this channel was incising down to this level (ca. − 3500 m) around 3.5 Ga or earlier (Supp. Figure 13).

**Table 1:** History of water discharge in Martian outflow channels.

Channel system	Initiation flooding activity	Subsequent/last flooding event
Ares Vallis	Late Noachian/Early Hesp. (ca. 3.6 Ga)^31^	Early Amazonian (ca. 2.5 Ga)^31^
Maja Valles	Late Noachian/Early Hesp. (ca. 3.7 Ga)^32^	Late Hesp/Early Amazon. (3.4–3.2 Ga)^34^
Mangala Region	Late Hesp/Early Amazon. (3.4–3.0 Ga)^21^	–
Marwth Vallis	–	Late Noach./Early Hesp. (3.5–3.7 Ga)^33^

Later episodes of channel activity have been reported in outflow channels within Mangala Region and Maja Valles during the Late Hesperian—Early Amazonian (ca. 3.4–3.2 Ga)^21,34^ (Table 1). Again, Mangala, Maja and Kasei Valles display a knickpoint zone around − 2500 m (Fig. 3) with identified shorelines^1^ (inferred from deltaic deposits at a similar elevation) dating to ca. 3.4 Ga^1,14,35^, thereby suggesting that these channels were incising down towards a common Northern ocean level at around − 2500 m (or slightly lower) by the late Hesperian.

Importantly, there are studies indicating volcanic activity and the volcanic resurfacing within Kasei Valles regions during the timeframe of our reconstruction^22–24^. For example, a volcanic cycle during the late Amazonian, consisting of several volcanic pulses including lava flows from Tharsis Montes and Echus Chasma that were identified in the base of channels < 1 Ga^23,24^. In our study, these lava flows are outside of the area we examined and the lava flows on the channel floor were not considered, as they do not meet our two criteria for crater counting (regarding counting area and similarity in geological history/crater density). Furthermore, there were two volcanic pulses in the Hesperian (3.4 ± 0.1 Ga)^22^, but only *one* of our surfaces (NX5-S2) contains areas identified as being formed by these volcanic episodes. We suggest that NX5-S2 is part of a terrace/surface (aligning along a common slope with other erosional surfaces) that formed at 3.50 ± 0.05 Ga, consistent with the formation of these other erosional surfaces in Kasei Valles (Fig. 1). Therefore, these Hesperian volcanic pulses either did not completely remove/obscure the crater population within NX5-S2 or occurred close in time with the second major flooding episode (3.5 Ga). To add weight to this argument, previous research suggested that these volcanic episodes postdate (though are close in time) the second flooding period in the Kasei Valles^22^.

The evolution of water on Mars is crucial to understand the past climatic/environmental conditions. Here, we show a unified record of past ocean levels interacting with channelized flows at different stages in Mars’ history. Importantly, our chronology data suggests that these flows were most likely episodic and limited in number—i.e. 35 dated surfaces within Kasei Valles imply the existence of 5 periods of channel flows/activity interacting with (at least) two past ocean levels, where the gaps (in time) between the common dated surfaces and the disequilibrium observed in the longitudinal profile^13^ suggest an ephemeral and/or episodic channel activity. Furthermore, this pattern of episodic flows emphasizes the major role of water on Mars during the Hesperian period (ca. 3.6–3.2 Ga), but also reveals that such activity—although potentially attenuated—continued into the early Amazonian (up to ca. 2.5–2.0 Ga). Thus, the environmental conditions still allowed (at least temporally) substantial amounts of liquid water running on the surface during this ‘cold and dry’ period^35^.

Additionally, our findings suggest that the Martian ocean rose in elevation (ca. 1,000 m) between ca. 3.6 Ga and ca. 3.2 Ga and soon afterwards disappeared, which implies a re-awakening of the Martian hydrosphere (in the Hesperian) re-occupying large portions of the Noachian Arabia shoreline^2,11^. Alternatively, the Martian ocean could have formed and dissipated episodically (as proposed in previous investigations^36^). Therefore, our results could have recorded the Deuteronilus ocean level during one episode (at ca. 3.6 Ga), after which the bulk of the Martian ocean became locked in cryospheric/subsurface deposits^36^ and returned to the Northern lowlands (up to the Arabia ocean level) during a subsequent episode around 3.4–3.2 Ga. Given the significant volumes of water required to induce this increase in ocean elevation^1,7^, it is unlikely that the water came solely from large episodic flood events. Therefore, we argue that additional sources of water, for example via groundwater release or the thickening of the Martian atmosphere^4,14^ were required. Furthermore, this increase in ocean elevation is also supported by a change from cryogenic-like climatic conditions^37^ towards a warmer climate permitting orographic precipitation-sourced runoff^4,35,38,39^ across the Hesperian. Thus, our results are consistent with a warmer and wetter early climate, which enabled the formation of a vast Hesperian ocean in the Northern Hemisphere and may have had profound implications on the potential habitability of the planet. Furthermore, they indicate a complex ancient Martian hydrosphere capable of supporting such an ocean, with an active hydrological cycle stretching into the Amazonian.

## Supplementary information

Supplementary Information 1.

Supplementary Information 2.
