# Supplementary material for: The Kasei Valles, Mars: a unified record of episodic channel flows and ancient ocean levels
Source: Sci Rep. 2020 Oct 29;10:18571. doi: 10.1038/s41598-020-75080-y (PMC7596472; doi:10.1038/s41598-020-75080-y)
Supplement: Supplementary file 1 — Supplementary Information 1. [file 41598_2020_75080_MOESM1_ESM.docx]

| The Kasei Valles, Mars: A unified record of episodic channel flows and ancient ocean levels |
| --- |

Sergio Duran^1*^ & Tom J. Coulthard^1^

^1*^School of Environmental Sciences, University of Hull, U.K. <S.Duran@2016.hull.ac.uk>

^1^School of Environmental Sciences, University of Hull, U.K. < T.Coulthard@hull.ac.uk >

# supplementary materials and methods

1. **Overview of methodology for reconstructing the evolution of Kasei Valles**

**This section describes the methodological approach adopted to reconstruct the valley evolution. This methodology follows four steps outlined below:**

1. **Selection of surfaces within Kasei Valles**
2. **Determination of the model age of each surface**
3. **Estimation of periods of cataclysmic activity**
4. **Cross-sectional and Longitudinal profile evolution**
   1. **Selection of surfaces within Kasei Valles**

**Definition of Cross-Sections (XSs)**

**In order to reconstruct the evolution of Kasei Valles, we initially defined nine cross-sections (XSs) all along both of its branches (Supp. Fig. 2). We intended to cover all surfaces of interest with the smallest number of cross-sections possible. We carried out the extraction of each XS on the global Mars HRSC MOLA Blended Digital Elevation Model (DEM) 200 m provided by the U.S. Geological Survey. The map projection is Simple Cylindrical and the total elevation uncertainty of the product is at least ± 3m. The DEM was imported in Arc-GIS, where the main trunk of the channel was defined. For this, we generated a sinkless raster by using the Arc-GIS filling functions, computed the direction of the flow and the accumulated flow. To define a drainage line, an empirically tested value of 1% of the maximum accumulated flow was used as a threshold to distinguish channel pixels from the background. The main trunk of the channel network was isolated. Subsequently, we plotted each XS roughly perpendicular to the main trunk of the channel by using the algorithms “Interpolate Line” and “Profile Graph” within the 3D Analyst tool. We included pre-flood highland surfaces at each end of the XS. By analysing the profile graph of each XS, we identified level surfaces at different elevations (Suppl. Fig. 1). These level surfaces could have had their crater population reworked by past megaflooding episodes in Kasei Valles. Hence, containing information on the timing of these episodes. Therefore, by using crater statistics, we can infer the time over which the crater population has been accumulating on these surfaces (date of formation or first exposure), as well as the last time they have been resurfaced (or when they were abandoned) by identifying when the crater population has experienced a minor modification. Hence, we considered all level surfaces identified within these XSs for further analysis. Finally, we visually inspected each level surface (by means of Mars Reconnaissance Orbiter high-resolution Context Camera (CTX) at 6m/pix and High Resolution Imaging Science Experiment (HiRISE) at 0.25m/pix imagery) to verify the presence of erosional and depositional landforms typical of flooding events. The 35 selected surfaces are shown in Sup. Fig. 2.**


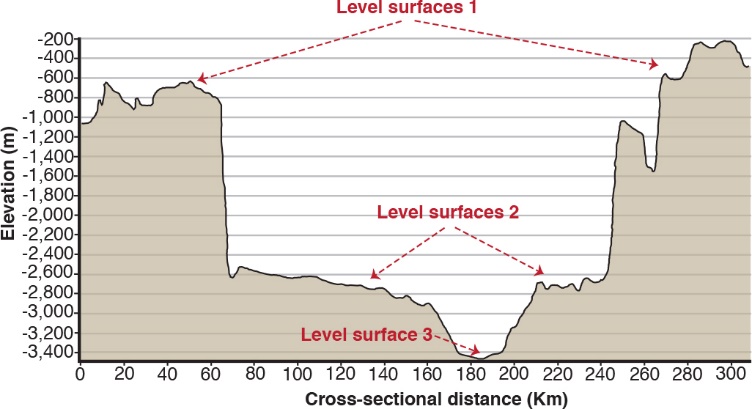


**Supp. Fig. 1. Identification of level surfaces within a given XS in Kasei Valles. The figure illustrates how level surfaces were manually identified within each XS in Kasei Valles. Level surfaces at similar elevations on both sides of the channel might share a common geological history. We used HiRISE and CTX imagery to further assess whether the crater population within both surfaces seems to be consistent with this hypothesis. When the populations were consistent, we included both within the counting locality boundary of this unit.**

**Additional Abandoned channels**

**The analysis of abandoned channels also performed in the present research. As for the level surfaces described in the previous section, by analysing these abandoned surfaces we can seek evidence for the time of first exposure/formation and last resurfacing/abandonment of surfaces within Kasei Valles. Hence, we incorporated these additional abandoned surfaces not captured by the XS analysis previously described. We constrained the analysis to those apparently abandoned trough-like surfaces (e.g. abandoned channels). Such abandoned surfaces are easy to detect since there is a significant difference in elevation between such surfaces and the lowest one within a same XS. In Arc-GIS, we imported the Mars HRSC MOLA Blended DEM Global 200m. We subsequently plotted a line perpendicular to both the main trunk (defined in the previous section) and the presumed abandoned surface. Then, we extracted the profile graph of the main channel and abandoned channel by using the algorithms “Interpolate Line” and “Profile Graph” within the 3D analyst tool. When the difference between the main trunk and the presumed abandoned surface was greater than 150 m, the surface was considered to be an abandoned surface. Likewise, surfaces within the area connecting the Northern and Southern branches in Kasei Valles, whilst not abandoned, were also included (Supp. Fig. 2). Finally, the presence of erosional and depositional landforms within each surface mentioned earlier was also verified by use of** **CTX imagery. Selected surfaces are shown in Supp. Fig. 2. A total of 35 surfaces within Kasei Valles were analysed.**


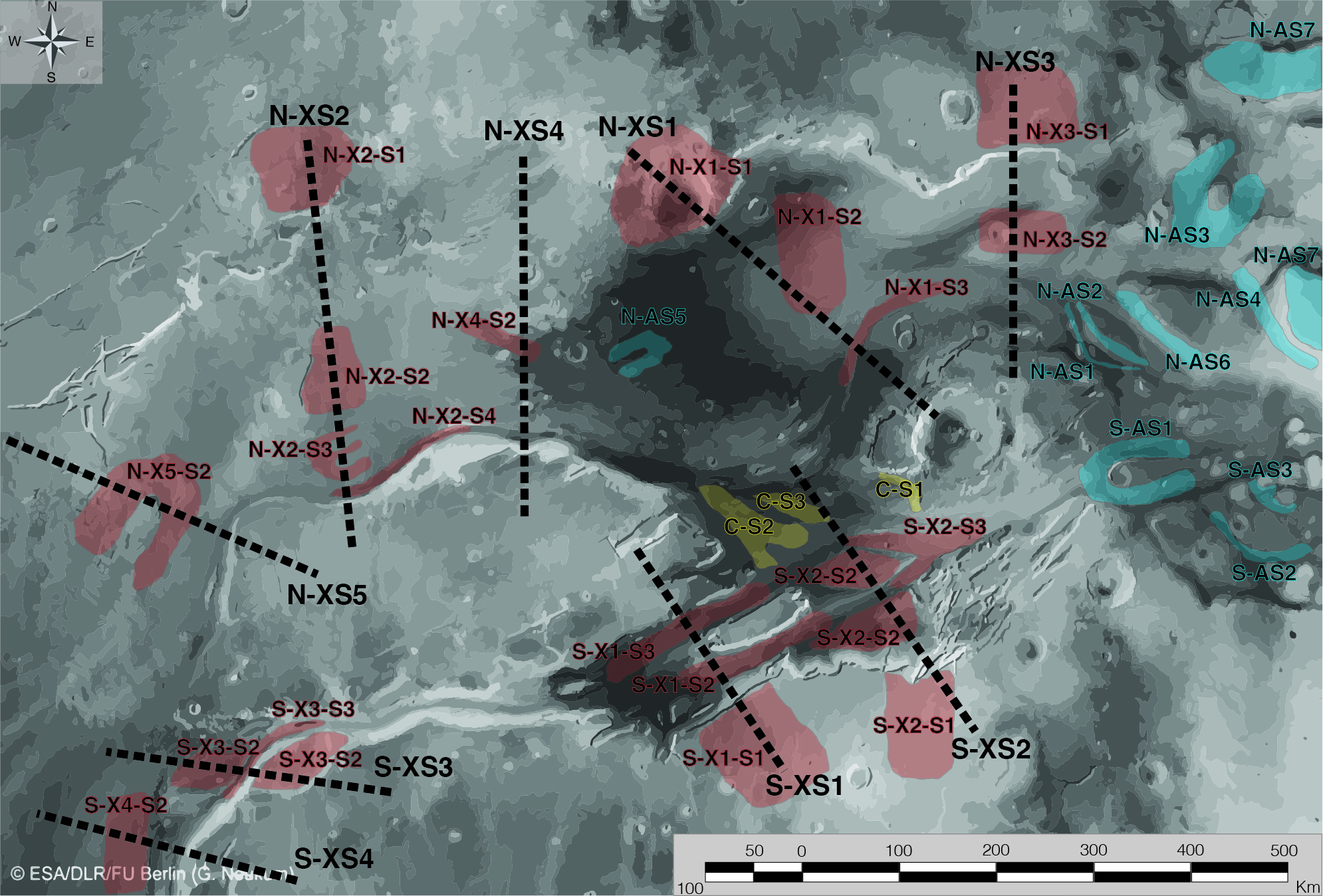


**Supp. Fig. 2. Surfaces used for subsequent crater size-frequency analysis in Kasei Valles. In red are those surfaces identified from XSs and in blue are surfaces identified as abandoned channels. The yellow colour indicates surfaces within the area connecting both branches. There are 35 surfaces analysed.** Credit: ESA/DLR/FU Berlin, CC BY-SA 3.0 IGO (<https://creativecommons.org/licenses/by-sa/3.0/igo/>). We modified this figure using Adobe’s Illustrator CS6 software (<https://www.ado-be.com/es/products/illustrator.html>) and Adobe’s Photoshop CS6 (<https://www.adobe.com/es/products/photoshop.html>).

- 1. **Determination of the model age of each surface**

**Background on the analysis of Crater Size-Frequency Distributions (CSFDs)**

**The analysis of crater size-frequency distributions (CSFDs) is a well-established and frequently applied method to determine surface ages of geological units on planetary bodies (Hartmann, 2005; Michael, et al. 2012). This method relies on mapping craters on planetary surfaces in order to infer the approximate time required to accumulate the observed size-frequency distribution of impact craters (Hartmann, 2005; Michael, et al. 2012). The methodology assumes that the given surface once existed in a pristine state with no superposed craters (Michael, et al. 2012). Over time, the surface accumulates impact craters: the older the surface, the more impact craters it should have (Michael, et al. 2012). As large impactors are less frequent than smaller ones, the older the surface, the larger the craters that are accumulated on it (Michael, et al. 2012). The methodology then associates the distribution of craters observed with an age by the use of production functions, which describes how many craters of a given size should form at a any given time period (Hartmann, 2005). Thus, the age of a planetary surface can be estimated by measuring the size-frequency distribution of superposed impact craters and then fitting this distribution to the production function for that body (Michael, 2013).**

**The CSFD method also allows us to assess the modification processes affecting a planetary surface (Hartmann, 2005). A resurfacing mechanism is one which process can remove/obscure the rims of a crater or infill it (Hartmann, 2005). Since smaller craters are more easily resurfaced than large ones, a resurfacing event causes a flattening of the crater size-frequency distribution by removing craters smaller than a certain diameter (d<D) (Hartmann, 2005). By fitting the flattened tail of the crater distribution to the production function, we can obtain the time required to re-accumulate these craters diameter (d) (Hartmann, 2005). Thus, the method also allows us to date the cessation of resurfacing activities on planetary surfaces (Hartmann, 2005).**

**To perform, we used CTX and** **HiRISE imagery in Arc-GIS. We then constructed map-projected CTX mosaics covering each of the surfaces selected in the section above. An Equidistant Cylindrical projection was applied to each mosaic. In total over 50,000 craters were identified and mapped.**

**Delimiting the counting area**

**The first stage of the CSFD analysis is to mark out the boundary of the area of interest within each surface. Selection of representative localities was required because (1) the total area and numbers of craters for each surface of interest were too large to perform CSFD analysis in a reasonable timeframe and (2) some surfaces contained areas that have inadequate CTX image coverage. We then used the extension CraterTools in Arc-GIS to draw a polygon with the boundary of the representative counting area.** **We delimitated representative areas that we interpreted to have a similar geologic history and excluded from them areas where we could identify significant deviations from this history (e.g. areas with steep slopes where craters would not accumulate at the same rate). In order to minimize the negative effect of pattern variability in cratering and the susceptibility of smaller craters to resurfacing (Warner, et al. 2015b), we used a minimum area of ca. 5,000 km^2^ and approaching to 10,000 km^2^ for each representative area. In cases where the representative area was smaller than 5,000 km^2^, we complemented our CSFD analysis with HiRISE imagery to understand whether our observations correspond to the most surficial unit or an underlying unit partly buried by a younger one. The minimum counting locality area considered was 900-1000 km^2^. Around 50% of the counting areas were higher than 5,000 km^2^ and around 90% higher than 1,000 km^2^ as shown in Table 2 (Appendix). We do not provide an in-depth analysis of the uncertainties that are inherent in the smaller area count statistics.**

**Extracting the crater population**

**The second stage of the procedure is to estimate the crater population within each representative area. We used CraterTools in Arc-GIS to define each crater using a 3 point digitalisation method. We mapped every identifiable crater within the counting locality, including those crater intersecting such area but with a geometric centre outside. We used a scale 1:18,000 to count craters, taking special care to identify only impact craters, thus trying to avoid other features as volcanic calderas or sublimation pits. All craters larger than 200m in diameter were included in our analysis. The reason of this threshold is that, in the case of analysing either (1) noisy CTX images or (2) noisy-free CTX but with impact structures heavily degraded, a minimum diameter larger than ca. 50m must be chosen to avoid resolution roll offs (Warner, et al. 2015b). In order to avoid these issues and minimize human errors, we selected a 200 m minimum diameter. We then changed the scale to 1:50,000 and 1:100,000 to ensure that the crater population was completely and accurately represented. Finally, we exported the counting area and the crater population into the software CraterStats.**

**Analyzing Spatial Randomness and Clustering**

**A random distribution of craters is defined by a normal distribution of crater diameters where any pattern on the surface could occur, but some patterns are more probable (Warner, et al. 2015b). The CraterStats software allows analysis of the spatial randomness of a crater population by (1) splitting the crater population into diameter bins and (2) performing a series of Monte Carlo simulations (Warner, et al. 2015b). In each iteration of these simulations, the counting locality is randomly scattered with a number and size of craters equivalent to those observed in a given bin (Warner, et al. 2015b). Over many iterations, the software develops a histogram describing the relative likelihood of each spatial distribution (Hartmann, 2005). The software repeats this process for every diameter bins, therefore obtaining a histogram per diameter bin (Hartmann, 2005). Each histogram shows the corresponding probability that the spatial pattern of the analysed diameter bin exhibits complete spatial randomness (Warner, et al. 2015b).**

**Subsequent to importing the counting area and crater population into the CraterStats software, we assessed whether our observed crater population has a representative random distribution. We performed a SDAA test with 300 iterations. Since we used relatively large counting areas (>5,000 km^2^), it is complicated to observe homogeneity over all the crater diameters. Commonly, clustering and ordering are observed in some crater diameters, but random distribution in others. For the purpose of our surface dating, we used a conservative 15 – 85% confidence interval for assigning randomness from the normal distribution. For values greater than ~ 85%, the pattern was considered clustered. Alternatively, for values smaller than ~ 15%, the pattern was considered ordered. We therefore excluded those diameter bins exhibiting clustered or ordered patterns for determining model ages.**

**Determining model ages**

**To determine model ages, the concept is to fit the observed CSFD of a surface to a known production function (Michael, 2013). In order to obtain an absolute age, the crater frequency for certain crater size bins is compared to a calibrated chronology function (Michael, et al. 2012). We used the production and chronology functions most commonly utilized with cumulative plots in the literature: the Ivanov (2001) and the Hartmann and Neukum (2001) respectively. We then employed cumulative plots and pseudo-log bin sizes to represent each CSFD. To obtain absolute ages, we used parts of the CSFD that (1) fit well to the production functions and (2) are comprised by a minimum of four bins of randomly distributed diameters. As CraterStats software allows for the fitting of multiple resurfacing ages at progressively smaller diameter craters (Hartmann, 2005), we determined the absolute age of first exposure and the absolute age of the last resurfacing period per counting locality if possible.**

**Applying a Resurfacing correction**

**We applied a resurfacing correction when the cumulative function curve fell beneath the base age isochronal and the resteepen again to nearly parallel a younger isochronal (fig. 3a). As craters at some diameters can be partly buried, the resurfacing correction allows us to account for the excess of large craters present in the cumulative function (Michael, et al. 2012). To apply the resurfacing correction, we considered only those randomly distributed larger diameter craters that fit well with the resurfacing correction to the younger isochronal (together with the randomly distributed smaller diameter already nearly parallel to this isochronal) (Suppl. Fig. 3b). As for the previous step, we considered a minimum of four bins of randomly distributed diameters to determine the absolute age. Since the direct output of the CraterStat is three significant digits (Warner, et al. 2015b), we reported all ages with this precision. We do not provide a treatment of uncertainties that are inherent to the development of the production and chronology functions for the Moon or Mars (Hartmann, 2005).**


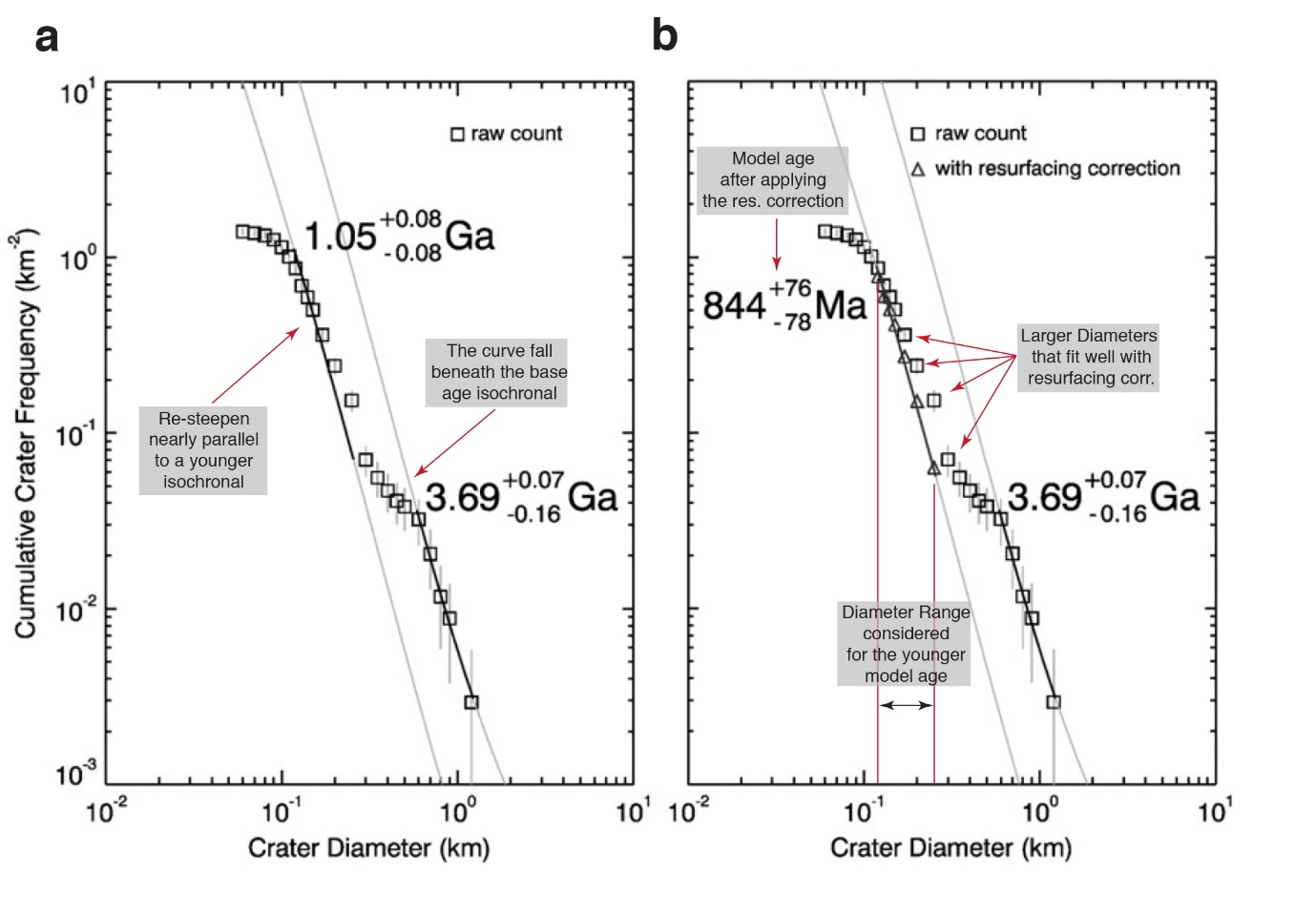


**Supp. Fig. 3. Example of a crater size-frequency distribution with a long-lasting resurfacing period. a: Determination model ages before applying a resurfacing correction. This image shows how we decided when to apply the resurfacing correction. b: determination model ages with resurfacing correction (the excess of older craters in the cumulative curve is removed, and a corrected younger model age of 844Ma is found). This image illustrates how we defined the diameter range for the resurfacing correction.**

**Classifying each absolute age as date of first exposure or last resurfacing**

**In the present research, we define *date of first exposure/formation* as being the period of time elapsed since craters started collecting on the surface, although the surface could be older if the evidence has been obscured. Alternatively, we define *date of last resurfacing/abandonment* as the time elapsed since a minor modification of the crater population occurred. For counting areas > 5,000 km^2^, we typically observed a tail of the CSFD comprised of large craters, a flattening on the slope of the CSFD probably caused by a resurfacing period(s) and a re-steepen of the slope of the CSFD towards smaller diameters. In these cases, we determined the absolute *date of first exposure/formation* from the largest population of craters (if they fit the production function well, as explained in section 1.2d) (Suppl. Fig. 4). We determined the absolute *date of last resurfacing/abandonment*** from diameters affected by the resurfacing process **(Suppl. Fig. 4). Occasionally,** more than one resurfacing period was observed. Thus, we defined *the* *date of last resurfacing* as the youngest model age determined from the range resurfaced diameters, if only minor subsequent modification in smaller diameters was observed.


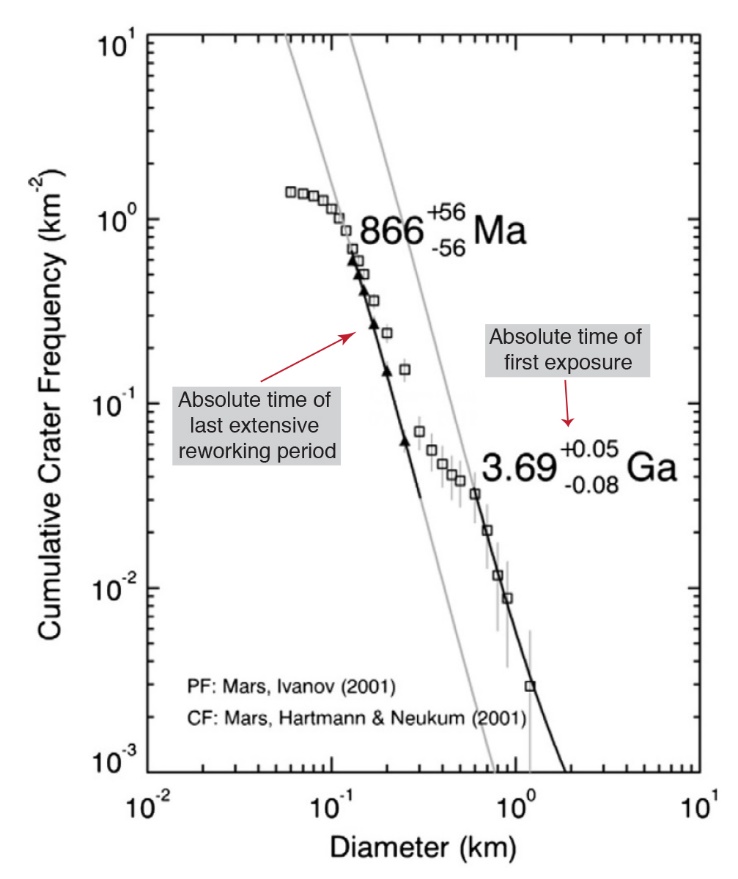


**Supp. Fig. 4. Another example of a given Crater size-frequency distribution with a long-lasting resurfacing period. The image illustrates how the absolute date of first exposure and the absolute date of last extensive reworking are identified within a large counting area (>5,000 km^2^)**

**In the case of counting areas < 5,000 km^2^, we needed to use CTX and HiRISE imagery to determine if our observations correspond to the most surficial unit or an underlying unit partly buried by younger one(s). We considered applying a resurfacing correction when performing the fitting to the youngest isochronal. The smaller the counting area, the less likely it is to contain large craters, thus the more likely it is to exhibit information about younger reworking periods (Warner, et al. 2015b).**

**Correlations in either the date of first exposure/formation or last resurfacing over separate surfaces may indicate that the same reworking mechanism was acting on these surfaces during the same time period. Therefore, this observation increases the likelihood of volcanism or fluvial erosion/deposition as a reworking agent. However, if the same mechanism has removed/obscured craters on separate surfaces, some systematic relationship between the diameters of craters affected and the removal/obscuration mechanism should exist. These relationships can give clues as to the process(es) underlying the removal/obscuration of craters (e.g. if volcanism were responsible, the thickness of the lava should decrease with distance from its source and, thereby, the diameters affected should also decrease). Thus, we finally compared the crater diameters affected by these temporal correlations. The Supp. Table 1 displays the counting localities selected, the branch in which each of them is located, the area of the counting locality and the absolute ages of date of first exposure and last reworking estimated.**

**Supp. Table 1. –** Database with the counting localities analyzed

| Counting Locality | Branch | Area (km^2^) | Absolute age of 1^st^ exposure (Ga) | Absolute age of last reworking (Ga) |
| --- | --- | --- | --- | --- |
| XS1-S1 | Northern Branch | 22,610 | ${3.70}_{-0.05}^{0.04}$ | ${3.50}_{-0.02}^{0.02}$ |
| XS1-S2 | Northern Branch | 8,324 | ${3.60}_{-0.09}^{0.05}$ | $-$^a^ |
| XS1-S3 | Northern Branch | 1,919 | ${3.50}_{-0.10}^{0.07}$ | $-$ |
| XS2-S1 | Northern Branch | 7,389 | $-$ | ${3.50}_{-0.09}^{0.05}$ |
| XS2-S2 | Northern Branch | 4,001 | $-$ | ${3.50}_{-0.04}^{0.03}$ |
| XS2-S3 | Northern Branch | 2,124 | $-$ | ${2.60}_{-0.50}^{0.40}$ |
| XS2-S4 | Northern Branch | 1,119 | $-$ | ${0.89}_{-0.10}^{0.10}$ |
| XS3-S1 | Northern Branch | 10,590 | ${3.90}_{-0.03}^{0.03}$ | $-$ |
| XS3-S2 | Northern Branch | 7,128 | $-$ | $-$ |
| XS4-S2 | Northern Branch | 2,243 | ${3.50}_{-0.10}^{0.07}$ | ${1.30}_{-0.08}^{0.08}$ |
| XS5-S2 | Northern Branch | 15,120 | ${3.50}_{-0.05}^{0.04}$ | $-$ |
| XS1-S1 | Southern Branch | 21,680 | ${3.60}_{-0.03}^{0.03}$ | ${2.30}_{-0.07}^{0.07}$ |
| XS1-S2 | Southern Branch | 6,189 | $-$ | ${2.00}_{-0.20}^{0.20}$ |
| XS1-S3 | Southern Branch | 3,168 | $-$ | $-$ |
| XS2-S1 | Southern Branch | 15,720 | $-$ | ${3.00}_{-0.10}^{0.10}$ |
| XS2-S2 | Southern Branch | 3,992 | ${3.50}_{-0.10}^{0.07}$ | $-$ |
| XS2-S3 | Southern Branch | 2,932 | $-$ | ${0.46}_{-0.06}^{0.06}$ |
| XS3-S2 | Southern Branch | 3,509 | ${3.50}_{-0.20}^{0.09}$ | ${2.00}_{-0.30}^{0.30}$ |
| XS3-S3 | Southern Branch | 1,262 | $-$ | ${0.69}_{-0.06}^{0.06}$ |
| XS4-S2 | Southern Branch | 8,533 | ${3.50}_{-0.10}^{0.06}$ | ${2.40}_{-0.20}^{0.20}$ |
| NAS1 | Northern Branch | 904 | $-$ | ${1.60}_{-0.10}^{0.10}$ |
| NAS2 | Northern Branch | 944 | $-$ | ${1.20}_{-0.20}^{0.20}$ |
| NAS3 | Northern Branch | 8,285 | $-$ | ${2.00}_{-0.20}^{0.20}$ |
| NAS4 | Northern Branch | 2,505 | ${3.50}_{-0.10}^{0.07}$ | ${1.80}_{-0.10}^{0.10}$ |
| NAS5 | Northern Branch | 5,065 | $-$ | ${2.40}_{-0.30}^{0.30}$ |
| NAS6 | Northern Branch | 2,759 | $-$ | ${2.30}_{-0.40}^{0.40}$ |
| NAS7 | Northern Branch | 6,490 | $-$ | ${3.30}_{-0.20}^{0.10}$ |
| NAS8 | Northern Branch | 7,686 | $-$ | ${3.20}_{-0.20}^{0.10}$ |
| SAS1 | Southern Branch | 6,226 | ${3.30}_{-0.50}^{0.10}$ | $-$ |
| SAS2 | Southern Branch | 3,777 | $-$ | ${2.10}_{-0.30}^{0.30}$ |
| SAS3 | Southern Branch | 1,011 | $-$ | ${2.90}_{-0.60}^{0.30}$ |
| CS1 | Connection N-S | 701 | ${3.20}_{-0.70}^{0.20}$ | ${1.10}_{-0.01}^{0.01}$ |
| CS2 | Connection N-S | 1,522 | $-$ | ${2.10}_{-0.50}^{0.50}$ |
| CS3 | Connection N-S | 1,821 | ${3.20}_{-0.20}^{0.01}$ | $-$ |

**^a^ No model age determined due to either not enough randomly distributed crater diameters in this part of the distribu-tion or a crater population insufficient to infer from older reworking periods**

- 1. **Determination of periods of channel activity**

**Grouping the data**

**In order to identify temporal correlations periods of first exposure/formation of surfaces and in periods of last resurfacing/abandonment, we assigned Kernel Density estimates to each calculated model age. In section 1.2f, we classified every absolute model age estimation as *date of formation* or *last resurfacing*. Here, we further classified each group depending on whether the representative area was in the *Northern* or *Southern* Branch of Kasei Valles. Therefore, we obtained four groups: (1) *date of first exposure/formation* of surfaces in the *Northern* Branch, (2) *date of first* exposure/formation of surfaces in the *Southern* branch, (3) *date of last resurfacing* of surfaces in the *Northern* branch and (4) *date of last resurfacing* of surfaces in the *Southern* branch.**

**Developing Gaussian Kernel Function per absolute model age estimate**

**For every absolute model age estimate for each counting locality, we used the best-fit age estimate and the associated uncertainty to develop a Gaussian Kernel Function for every age estimate. The bandwidth correspond to the uncertainty of the estimate and the amplitude equals 1. For asymmetrical uncertainties, the Kernel functions consisted of the combination of two Gaussian Kernel Functions, one for each side (Supp. Fig. 5). To generate the Gaussian Kernel Functions we used the function “Norm.Dist()” in Excel. We defined each value within the Gaussian by calculating the distance between that best fit and the corresponding age, and subsequently assigning to that value the corresponding normal distribution (“Norm.Dist()” ) with a mean equal to zero and the standard variable (SD) worked out by equation 1:**

**SD = Uncertainty/3.45 (1)**

**The equation was empirically tested, as well as generates frequencies < 0.005 at the boundaries and 1.000 at the best-fit (Supp. Fig. 5). At the end of this stage, we had a Gaussian Kernel Function per age estimate with a density value equal to one at the best-fit and zero at the boundaries (Supp. Fig. 5).**


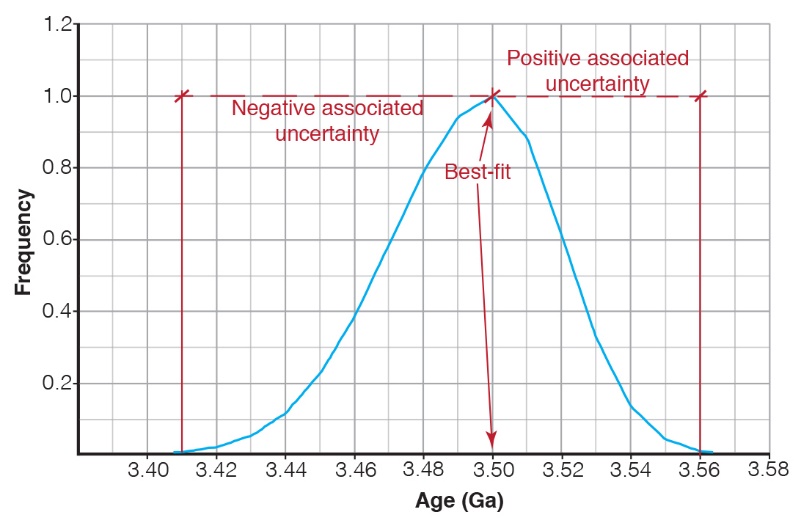


**Supp. Fig. 5. Asymmetrical Gaussian Kernel Function generated for a model age estimate with a best-fit equal to 3.5Ga, negative uncertainty equal to 0.09Ga and positive uncertainty 0.06Ga. The image illustrates how we defined asymmetrical Gaussian Kernel Functions per each absolute age estimate.**

**Developing a Kernel Density Estimate per group**

**Ultimately, we developed a summed Kernel Density estimate for each of the groups (1 - 4) previously defined in the section 1.3a. We summed (per age) the individual Kernel Function values within the same group. The Kernel Density Estimates were presented in two different graphs: (1) First exposure/Formation and (2) Last Resurfacing/Abandonment. Each graph therefore contains the *Kernel Density Estimate* corresponding to the *Northern* Branch and the analogue corresponding to the *Southern Branch*, as well as the *Total Kernel Density estimate* representing the sum of the two individual Kernel Density estimates. The Supplementary figure 6 shows the graph corresponding to the absolute age of first time of exposure. We defined a commonality as a period in which the Total Kernel Density estimate has a value greater than one. As stated in the section 1.2f, such temporal correlations should identify periods during which a given mechanism was reworking more than one surfaces.**

**
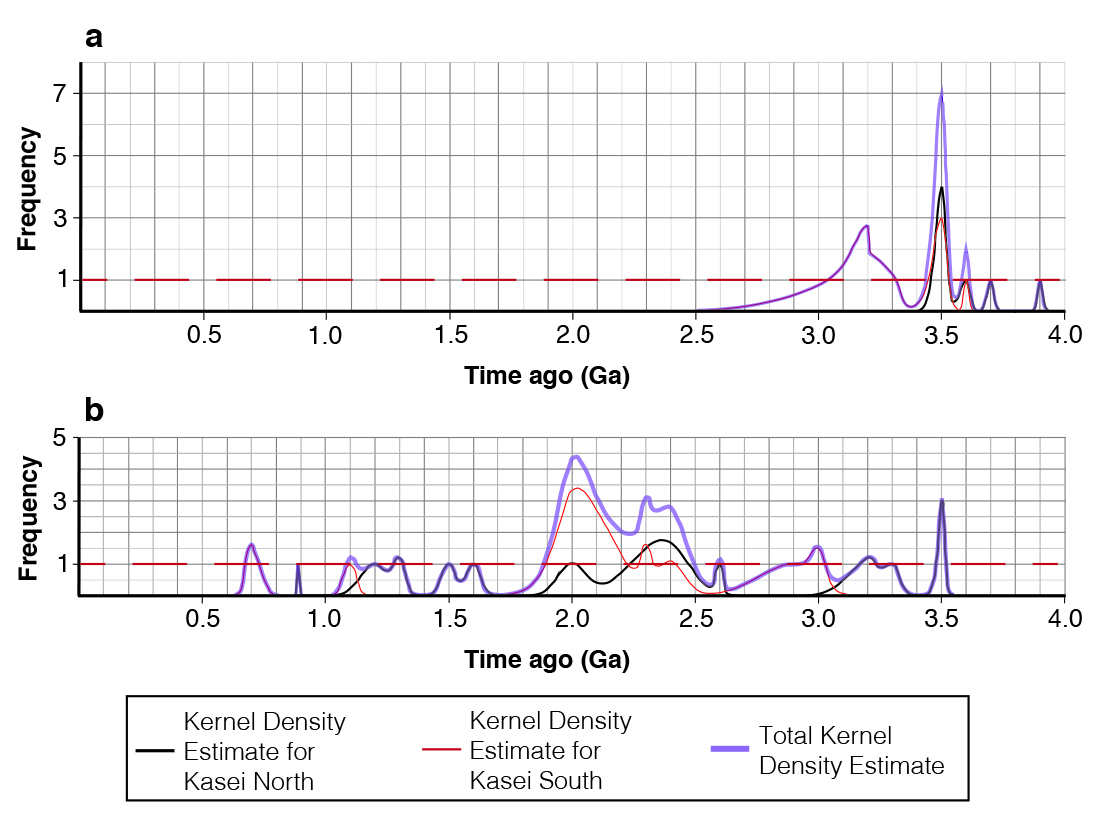
**

**Supp. Fig. 6.- Kernel Density estimates displaying the frequency of the occurrence of absolute ages of the dated surfaces in Kasei Valles.** The graphic “a” displays the Kernel Density Estimates describing the frequency of surfaces formed/first-exposed over time. The graphic “b” displays the Kernel Density Estimates describing the frequency of surfaces abandoned/last-reworked over time.

- 1. **Cross-Sectional and Longitudinal profile evolution**

**Extracting the longitudinal profile of both branches**

**The extraction of the longitudinal profile of the main trunk of both branches in Kasei Valles was carried out on the Mars HRSC MOLA Blended DEM Global 200m provided by the U.S. Geological Survey. We first imported this DEM into Arc-GIS, where channel networks were extracted. For this, we generated a sinkless raster by using Arc-GIS filling functions, computed the direction of the flow and the accumulated flow. To define a proper drainage line, an empirically tested value of 1% of the maximum accumulated flow was used as a threshold to distinguish channels. The main trunk of both branches was isolated, defining the topographic elevation -3,750m as the bottom end of the channel. To prepare the main trunk of the channel to be exported and to aid subsequent tests, we split the channel into a 3km reaches. We added a point at the end of each reach and, finally, we extracted the elevation all of these points from the sinkless DEM to generate the channel longitudinal profiles. We conducted the same procedure for the main trunk in both branches. We created a table with the number of the reach, the initial and final elevation and its distance upstream (km). By plotting the elevation of each reach against the upstream distance, we obtained a representation of the longitudinal profile of both branches.**

**Extracting the longitudinal profile within each counting locality**

**The extraction of the longitudinal profile within the counting localities considered in the present work was also carried out on the Mars HRSC MOLA Blended DEM Global 200m provided by the U.S. Geological Survey. We imported the DEM and the polygon shape of the counting locality into Arc-GIS. As occasionally Arc-GIS generated unrealistic channel networks over these areas, we generated a polyline in Arc-GIS following the most-likely trajectory of the water for these cases. In order to do that, we generated a polyline in Arc-GIS following such trajectory. To prepare the polyline to be exported and to aid subsequent tests, we split it into a 3km reaches. We added a point at the end of each reach and, finally, we extracted the elevation of all these points from the sinkless DEM (obtained earlier). We subsequently generated the channel longitudinal profiles from these points. We conducted the same procedure for all the counting localities analysed. To plot all longitudinal profiles in a same graph, we needed to calculate the distance that the water travelled to reach each counting locality. Thus, we defined a polyline in Arc-GIS broadly representing the trajectory of the water from the most upstream part (Supp. Fig. 7 & 8). To define a representative trajectory, we assumed that the counting locality was part of the active channel bed (therefore, we assumed that surfaces at lower elevations were created later on). We subsequently measured the length of this polyline and added such value to the distance upstream of each reach of the longitudinal profile within the counting locality (Supp. Fig. 8). We created a table with the number of the reach, the initial and final elevation and its distance upstream (km). By plotting the elevation of each reach against the upstream distance, we obtained a representation of the longitudinal profile within each counting locality.**

**
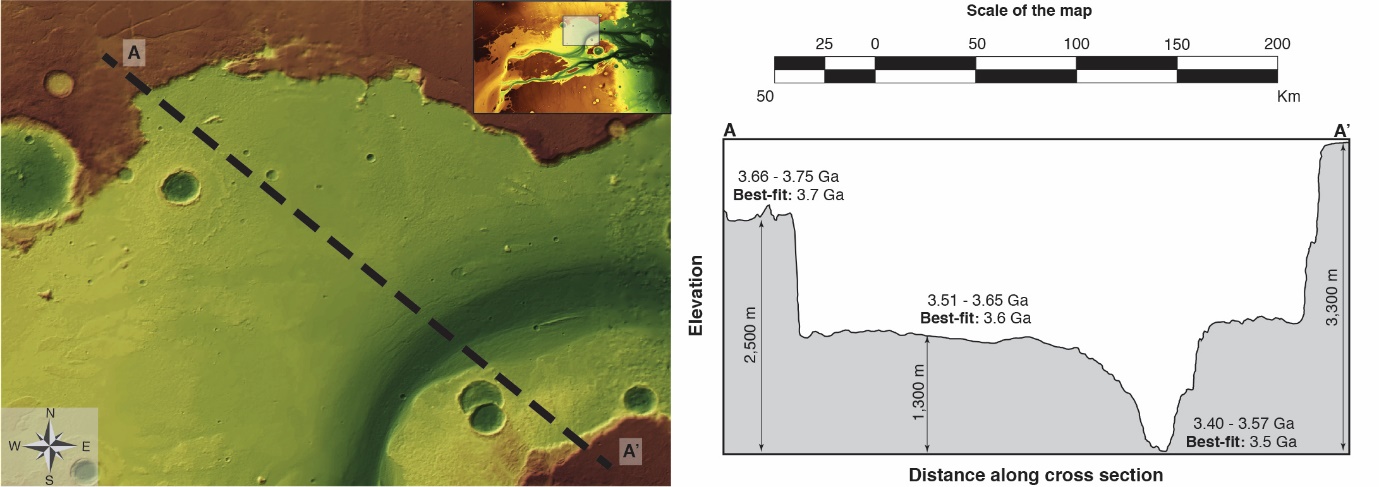
**

**Supp. Fig. 7.- Cross-sectional (XS) of Kasei Valles.** On the left, a top-down view of the XS selected with an inset panel showing the location of this view within Kasei Valles. On the right, the XS annotated with the estimated absolute model ages of the surfaces within it. This provides an estimate of the total amount of time required to carve this XS. We produced this figure using Adobe’s Illustrator CS6 software (<https://www.ado-be.com/es/products/illustrator.html>).


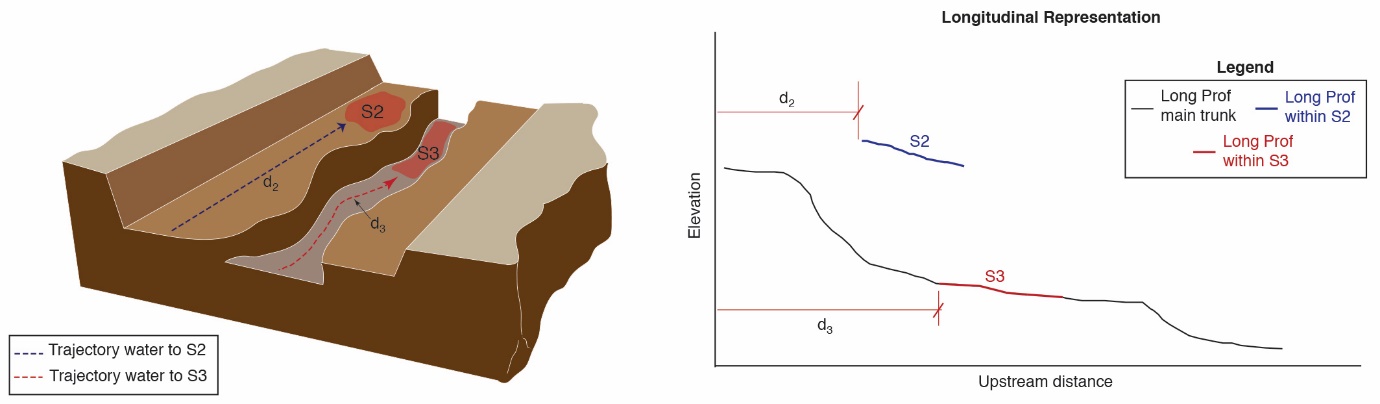


**Supp. Fig. 8. Schematic diagram showing how we defined representative trajectories of water towards different counting localities, as well as how we represented the longitudinal profile within each counting locality on the same graph. On the left, it shows how a representative waterpath is defined. To do that, we assumed that the counting locality was part of the active channel bed (therefore, we assumed that surfaces at lower elevations were created later on). On the right, it illustrates how different longitudinal profiles within different counting localities are represented in a same graph.**

**Assessing the Cross-sectional and longitudinal profile evolution**

**To analyse the evolution of the longitudinal profile over time, we plotted two different graphs. The first graph shows the longitudinal profile of the current thalweg and the longitudinal profile of all surfaces from which we know the date of first exposure/formation. The second graph shows the longitudinal profile of the current thalweg and the longitudinal profile of all surfaces from which we know the date of last resurfacing/abandonment. We coloured all surfaces formed during the same period with the same colour. Likewise, we coloured all surfaces abandoned during same period with the same colour. We assigned these colours based on the temporal correlations calculated in the section 1.3c. The small number of temporal correlations observed in last resurfacing younger than 2.0 Ga (fig. 2 in the manuscript) were not considered to be related to fluvial activity and not assigned any color, since they have been inferred from significantly smaller diameters (Table 2 - Appendix) – thus more susceptible to be affected by other erosional agents such as aeolian activity – and do not appear to follow any relationship with elevation, which reduces the likelihood that they are caused by fluvial erosion and suggest other resurfacing mechanisms (e.g. aeolian activity).**

**An analysis of the formation and evolution of the longitudinal profile allows for inferring about elevations towards which the valley was grading down during different periods. Further, an analysis of the abandonment of different longitudinal profiles allows us to infer which surfaces were active during different periods of megaflooding activity. The observation of surfaces formed at lower elevations, while other abandoned ones exist at higher elevations within a same region, gives evidence for vertical incision caused by flooding activity. Thus, a period of extensive formation of surfaces that also exhibits extensive abandonment of higher-elevated surfaces further indicates fluvial/flooding activity as the responsible mechanism. Finally, we assigned the same colours to the corresponding surfaces within the XSs generated in the section 1.1a.**

1. **Additional analysis on other Martian channels and valleys**

**To support the inferences made from the dated surfaces and channels, we also widened the scope of this stude to include two other channel systems: Mangala Valles and Ares Vallis. Previous research (Neukum, et al., 2010; Warner, et al., 2010) has dated some channel activity during the periods we are considering and we thus aim to expand out methods to these areas. Thereby comparing these results to our findings from Kasei Valles.**

- 1. **Estimating formation time of the base-level change knickpoints in Ares Vallis**

**One base-level change knickpoint at ca. -3,500m has been already detected in Ares Valles^10^. Due to the lack of counting area within the strath terraces formed as the knickpoint retreated, we selected another larger strath terrace located at a higher elevation but in the same area to analyse (Supp. Fig. 9). The reasoning is that if such surface has been abandoned, the last resurfacing time could be related to the pulse of vertical incision forming this knickpoint. Therefore, the last resurfacing time marks a maximum age for the formation of the knickpoint. In other words, this knickpoint probably formed at either the time of abandonment of this surface or later on. To define the counting area and perform the age estimate, we followed the same methodological approach to that described in section 1.2.**


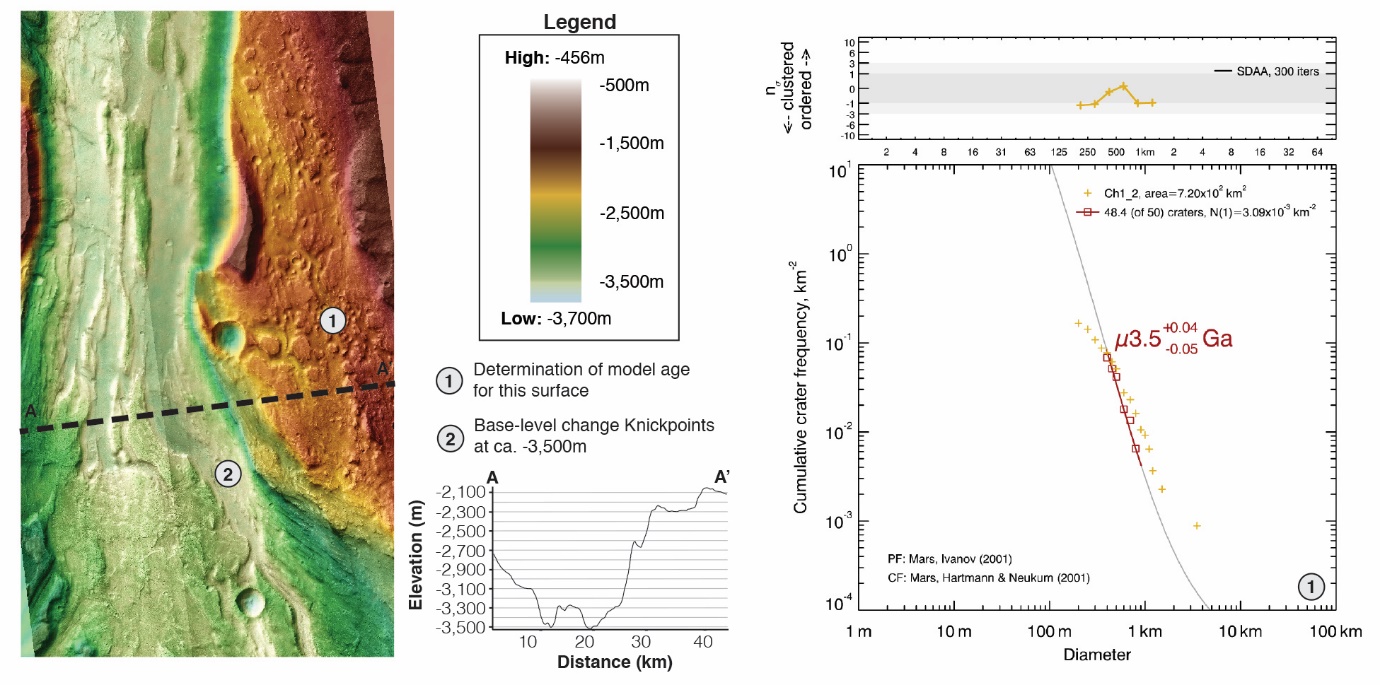


**Supp. Fig. 9. Estimating the formation time of the base-level change knickpoint at ca. -3,500m within Ares Vallis. As strath terraces and the surface reworked as this knickpoint retreated do not give us enough area to perform a reliable counting analysis, the surface one was selected and the abandonment of such surface was related to the formation of this knickpoint (illustrates as two in the figure).** We produced this figure using Adobe’s Illustrator CS6 software (<https://www.ado-be.com/es/products/illustrator.html>).

- 1. **Estimating when the Mangala region was formed**

**The Mangala Valles region contains channels with base-level change knickpoints at about -2,500m (Duran, et al. 2019). In addition, previous investigators found that these channels were formed during one period of intense flooding activity towards the Late Hesperian (Neukum, et al. 2010). Thus, elucidating whether this region formed in the Late Hesperian can give further support to the presence of a global body of water at ca. -2,500m in this period.**

**Extraction longitudinal profiles of Mangala Valles, Abus Valles and Mangala Valles**

The longitudinal profile of a channel gives geomorphological evidence of its fluvial history. If the Mangala region was actually carved when a body of water was present in the Northern lowlands, these longitudinal profiles could have recorded evidence of such a body of water. We therefore extracted longitudinal profiles of Mangala Valles for further interpretations. We followed the procedure described in the section 1.4a to extract these longitudinal profiles.

**Period/s of cataclysmic activity in Mangala region**

To assess whether base-level change knickpoints in the Mangala Valles region were formed towards the Late Hesperian, we have analyzed the crater population within a region in Mangala Valles (Supp. Fig. 10). We selected a counting locality within the floodplains of Mangala Valles, since it provides a significant area and sufficient crater population to analyze. Furthermore, such location should allow us to infer how many reworking periods affected these floodplains, as well as estimate the absolute model age for such flooding activity. If only one period of cataclysmic activity is observed, these knickpoints may have been formed during such period. We followed the same procedure as detailed in section 1.2 to select the counting area and determinate the absolute model age.


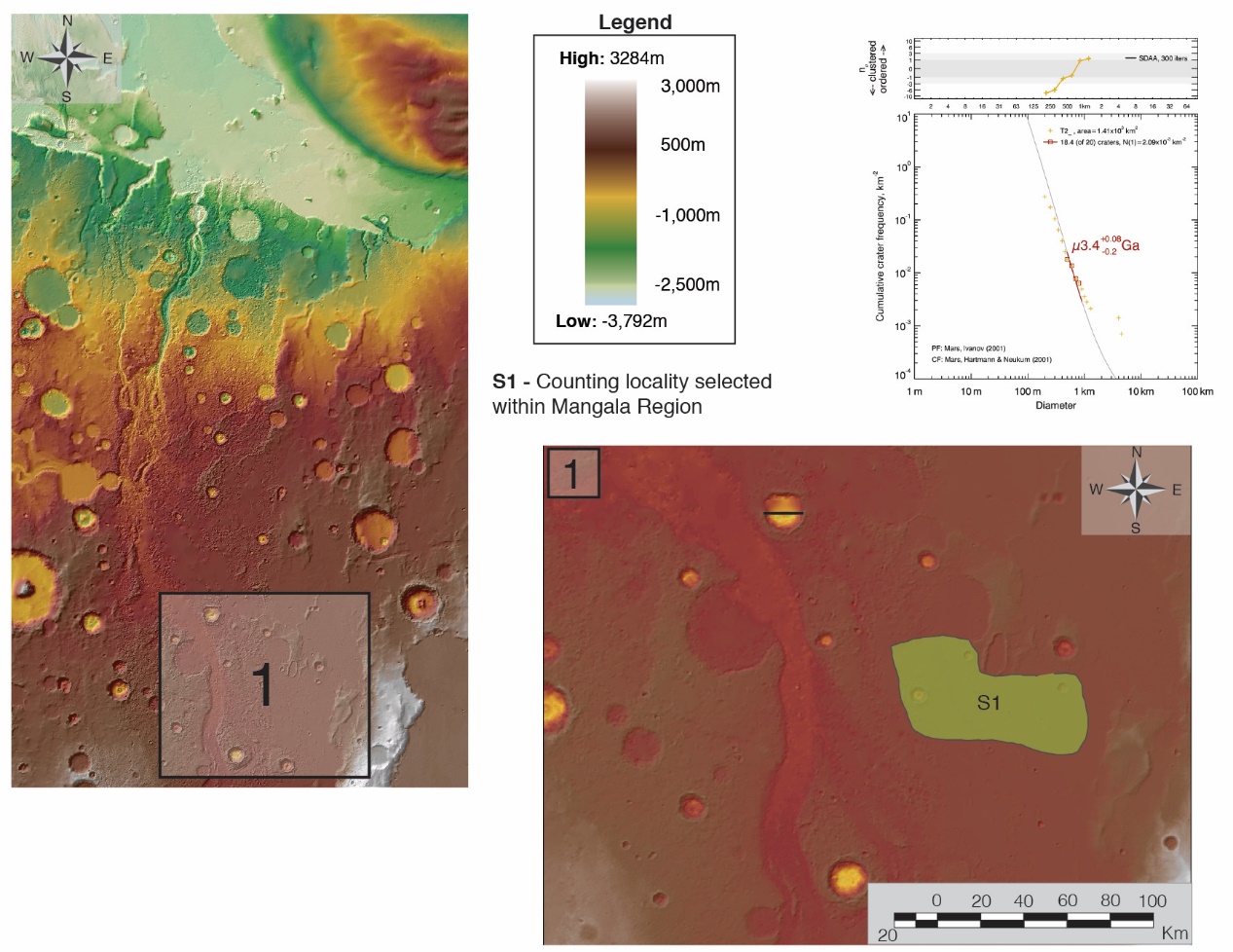


**Supp. Fig. 10. Estimating the formation time of Mangala Valles. The image on the left shows a map of the region of Mangala System, including Mangala Valles, Minio Valles and Abus Valles. Additionally, it shows with the rectangle number one the region subjected to further analysis. The image on the right illustrates where the counting area is located.**

**REFERENCES CITED**

- Hartmann, W.K. (2005). Martian cratering 8: Isochron refinement and the history of martian geologic activity. *Icarus* **174**, 294–320.
- Michael, G.G.; Plazt, T.; Kneissl, T.; and Schmedemann, N. (2012). Planetary surface dating from crater size–frequency distribution measurements: Spatial randomness and clustering. *Icarus* **218**, p. 169-177.
- Warner, N.H., Gupta, S., Calef, F., Grindod, P., Boll, N. & Goddard, K. (2015). Minimum effective area for high-resolution crater counting of Martian terrains. *Icarus* **245**, p. 198-240.

**ADDITIONAL FIGURES**

**
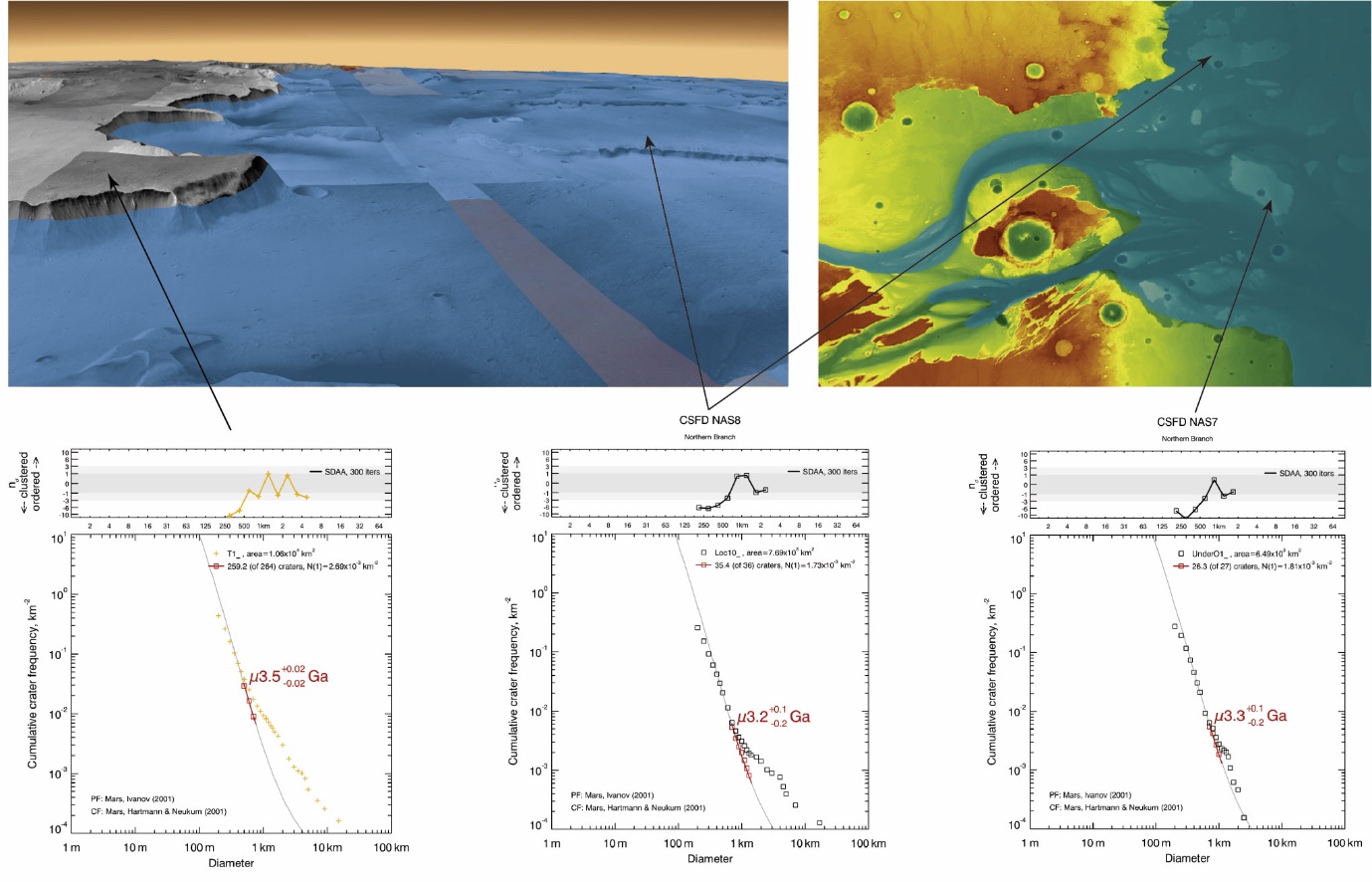
**

**Supp. Fig. 11.- Resurfacing shown by the crater population of high-elevation areas which could have been facilitated by an increase in base-level up to ca. -2,500m.** The image on the left shows a 3D view of the mouth of Kasei Valles, in which the blue colour correspond to a simulated ocean at ca. -2,500m elevation. The image shows high-elevation surfaces (about 1,000m higher than the channel bed) at the mouth of Kasei Valles become inundated. This could explain facilitated the resurfacing of crater population within these high-elevation areas (this signal can be seen in the CSFD plots in the center and on the right). Surfaces above -2,500m do not exhibit this resurfacing signal, as shown by the CSFD plots on the left. We produced this figure using Adobe’s Illustrator CS6 software (<https://www.ado-be.com/es/products/illustrator.html>) and Adobe’s Photoshop CS6 (<https://www.adobe.com/es/products/photoshop.html>).

**
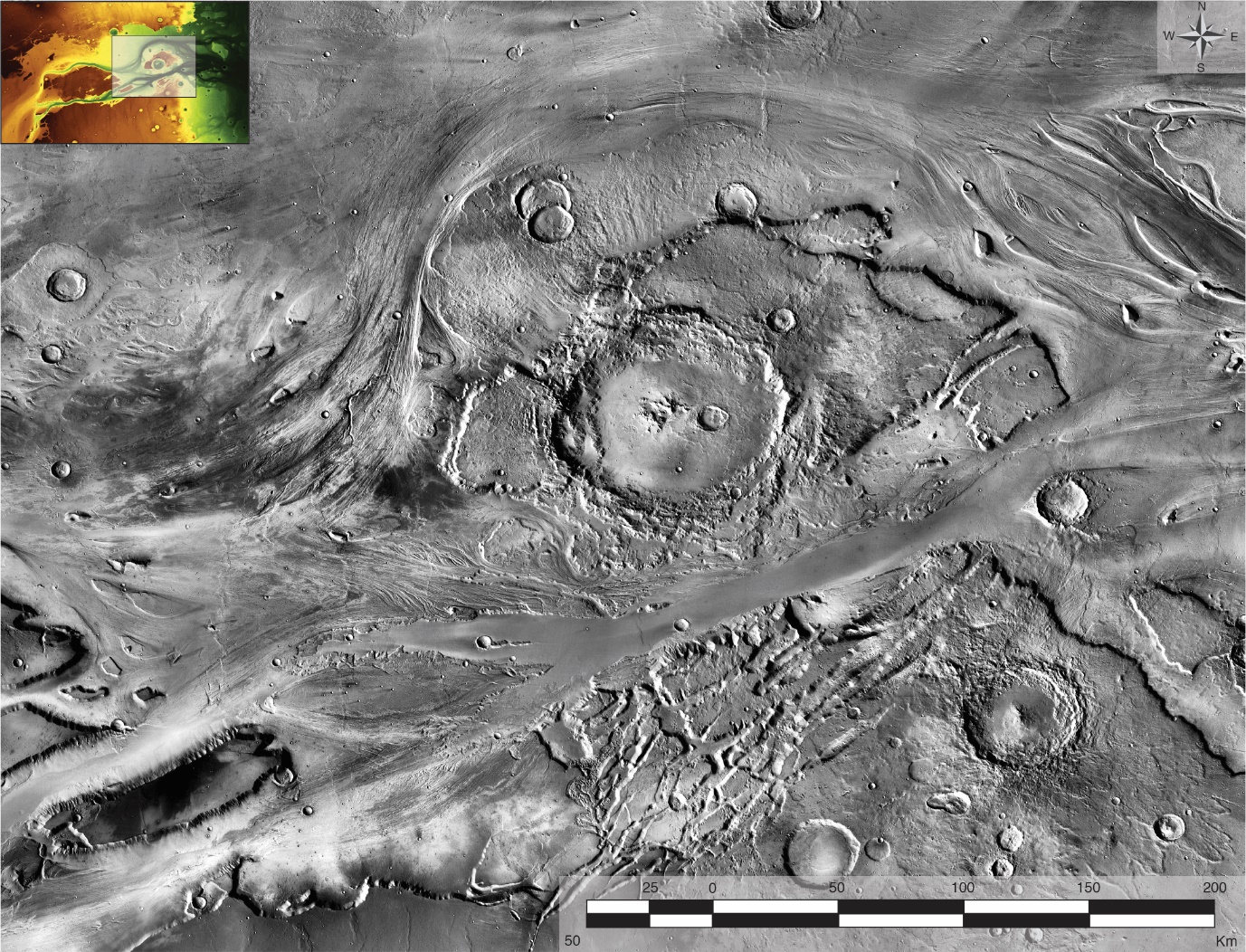
**

**Supp. Fig. 12.- THEMIS image showing the mouth of Kasei Valles.** The image displays both branches separated by the Sharonov Crater. In the Southern branch, there is a knickpoint. The headward erosion (leading to this knickpoint) probably triggered an episode of river capture connecting both branches at around 3.0-3.2 Ga ago. Whilst less pronounced, the Northern branch also exhibits a knickpoint at the same level. Image credit: THEMIS 059, THEMIS Public Data Releases <http://themis-data.asu.edu>. We modified this image using Adobe’s Illustrator CS6 software (<https://www.ado-be.com/es/products/illustrator.html>) and Adobe’s Photoshop CS6 (<https://www.adobe.com/es/products/photoshop.html>).


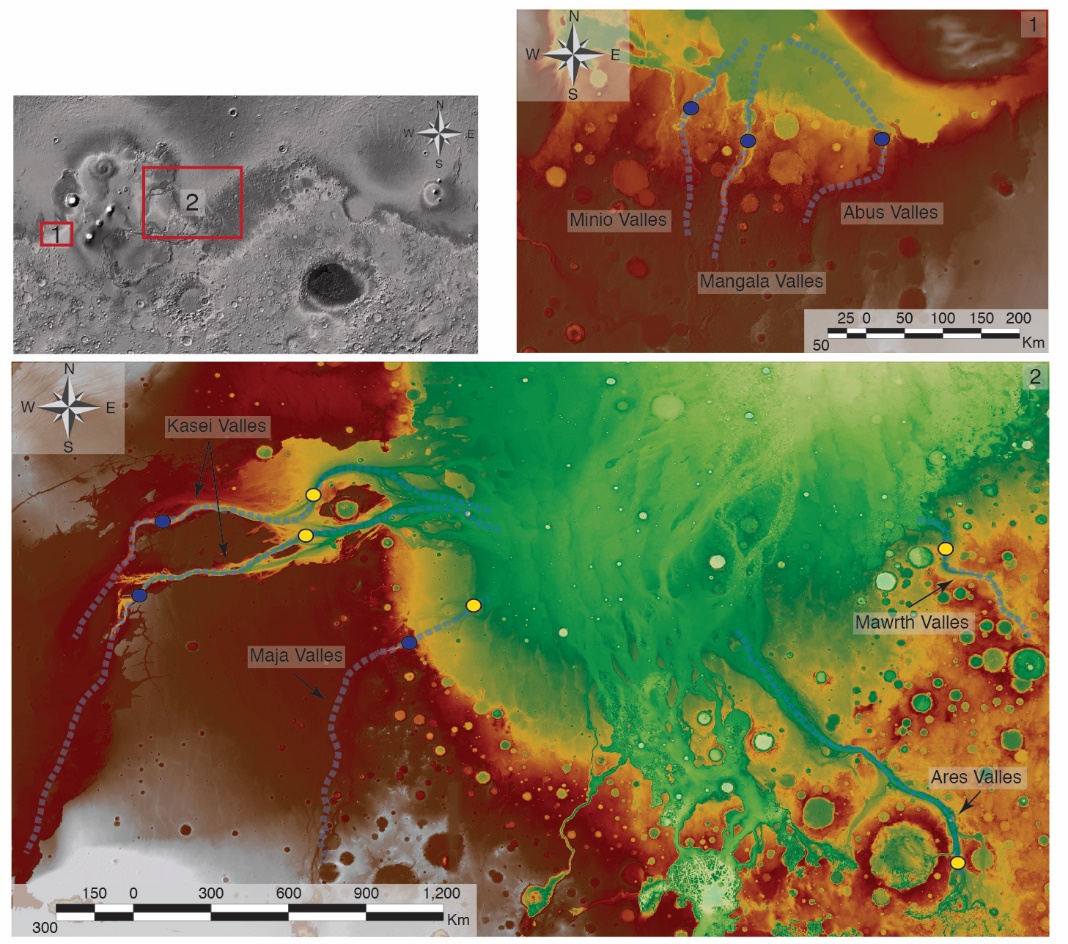


**Supp. Fig. 13. Location of the 10 base-level change knickpoints used in the present research. Blue circles indicate the locations of base-level change knickpoints at elevations ca. -2,500m. Alternatively, yellow circles indicate the locations of base-level change knickpoints at ca. -3,500m. The figure also contains the name of the channel system, a scale bar and a global map displaying the location of each sub-figure.** We produced this figure using Adobe’s Illustrator CS6 software (<https://www.ado-be.com/es/products/illustrator.html>).


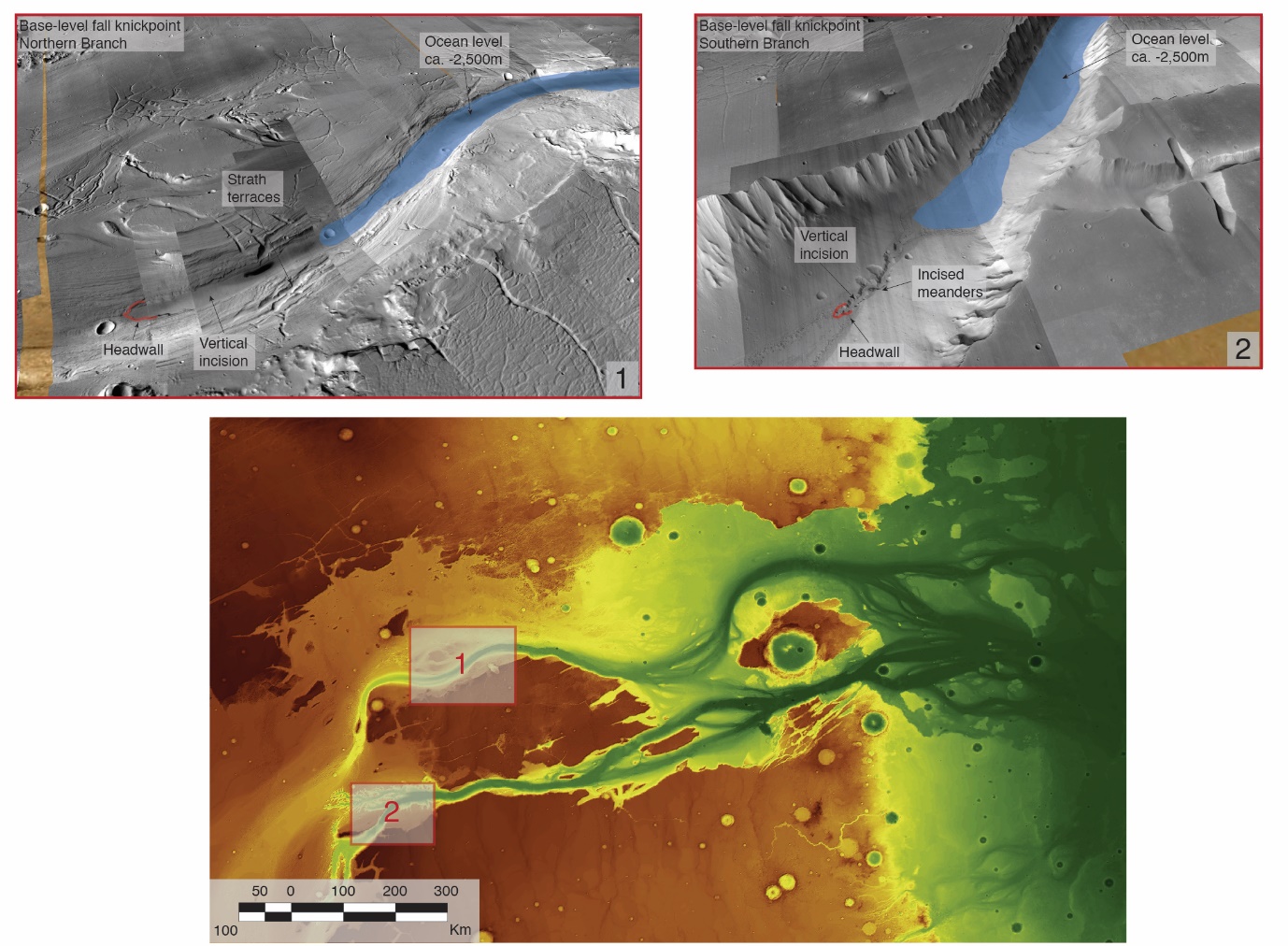


**Supp. Fig. 14. Aerial sketched images of the base-level change knickpoints at ca.-2,500m elevation in Kasei Valles . Aerial HiRISE and CTX images of these knickpoints. Each image was sketched with the detected features that a base-level change knickpoint typically exhibits. A map of Kasei Valles illustrates the location of each Aerial image.** We produced this figure using Adobe’s Illustrator CS6 software (<https://www.ado-be.com/es/products/illustrator.html>) and Adobe’s Photoshop CS6 (<https://www.adobe.com/es/products/photoshop.html>).


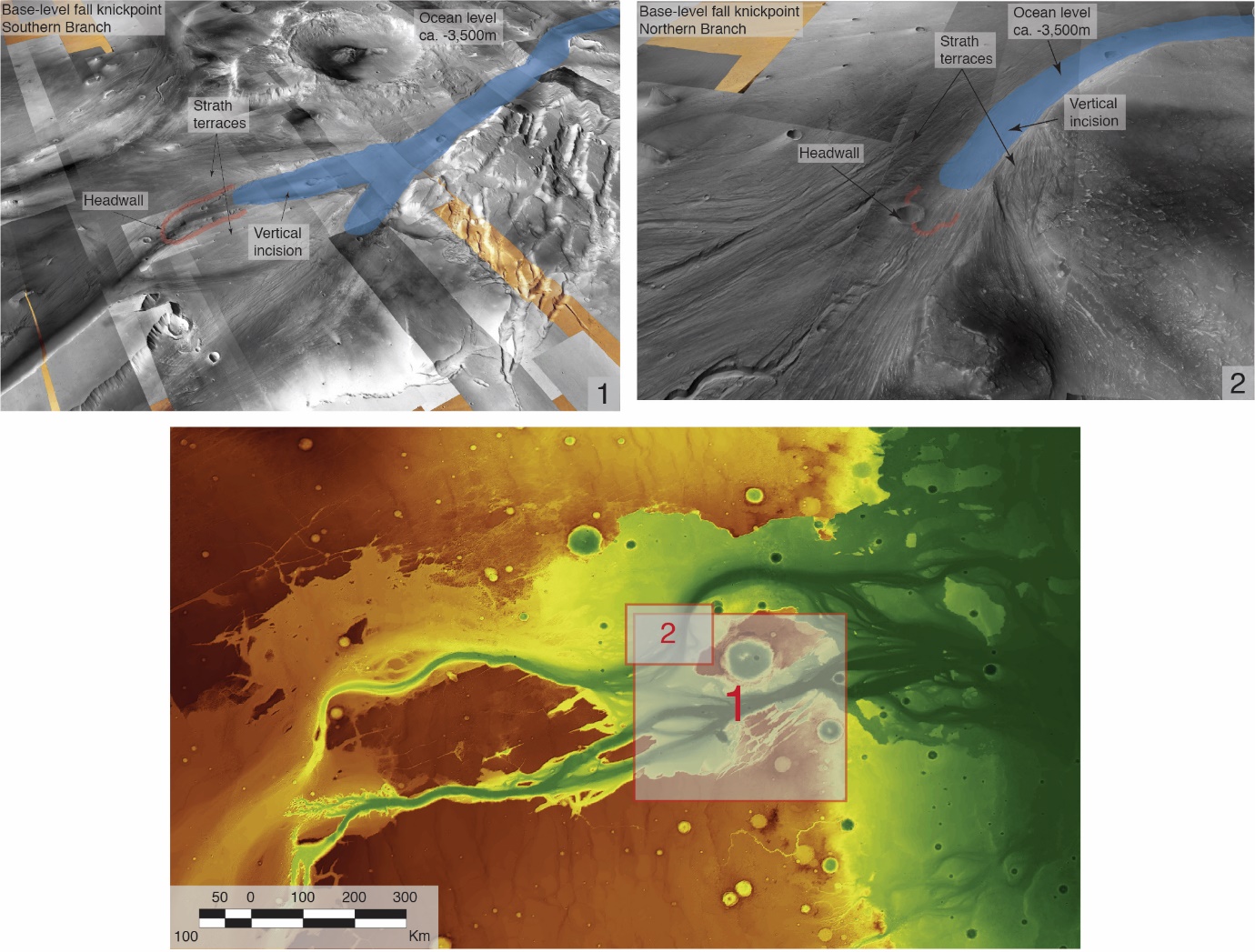


**Supp. Fig. 15. Aerial sketched images of the base-level change knickpoints at ca.-3,500m elevation in Kasei Valles . Aerial HiRISE and CTX images of these knickpoints. Each image was sketched with the detected features that a base-level change knickpoint typically exhibits. A map of Kasei Valles illustrates the location of each Aerial image.** We produced this figure using Adobe’s Illustrator CS6 software (<https://www.ado-be.com/es/products/illustrator.html>) and Adobe’s Photoshop CS6 (<https://www.adobe.com/es/products/photoshop.html>).


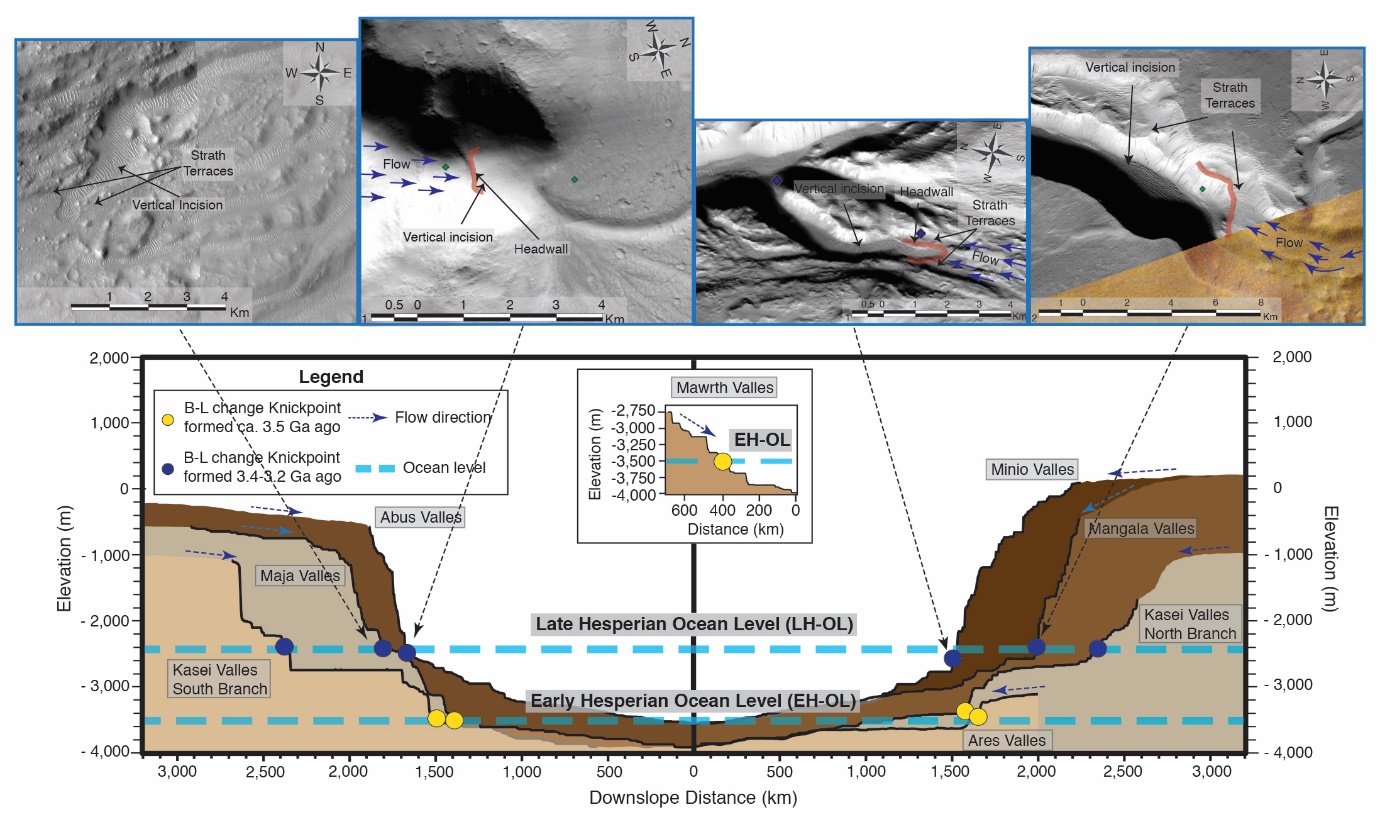


**Supp. Fig. 16. Aerial sketched images of the base-level change knickpoints at ca.-2,500m elevation in other outflow channels. Aerial HiRISE and CTX images of these knickpoints. Each image was sketched with the detected features that a base-level change knickpoint typically exhibits. A schematic representation illustrates the location of each knickpoint.** We produced this figure using Adobe’s Illustrator CS6 software (<https://www.ado-be.com/es/products/illustrator.html>).


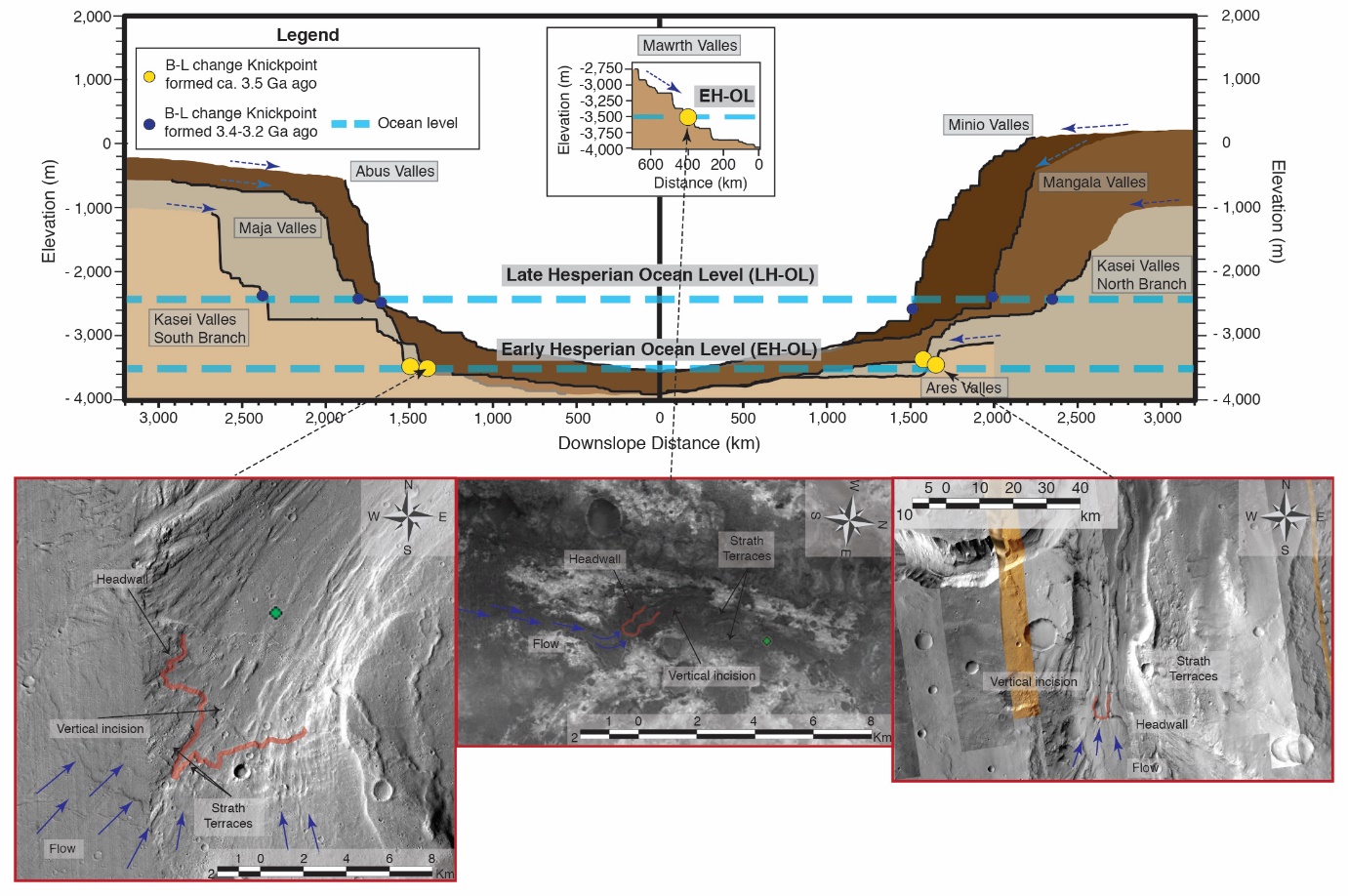


**Supp. Fig. 17. Aerial sketched images of the base-level change knickpoints at ca.-3,500m elevation in other outflow channels. Aerial HiRISE and CTX images of these knickpoints. Each image was sketched with the detected features that a base-level change knickpoint typically exhibits. A schematic representation illustrates the location of each knickpoint.** We produced this figure using Adobe’s Illustrator CS6 software (<https://www.ado-be.com/es/products/illustrator.html>).
