# Supplementary material for: The Kasei Valles, Mars: a unified record of episodic channel flows and ancient ocean levels
Source: Sci Rep. 2020 Oct 29;10:18571. doi: 10.1038/s41598-020-75080-y (PMC7596472; doi:10.1038/s41598-020-75080-y)
Supplement: Supplementary file 2 — Supplementary Information 2. [file 41598_2020_75080_MOESM2_ESM.docx]

**Appendix**

**Model age estimates for surfaces within Kasei Valles**

**Table 2.** Assigned model ages from 35 representative counting localities analyzed in the present research

| Counting Locality | Branch | Area (km^2^) | Absolute age of 1^st^ exposure (Ga) | | | | | | | Absolute age of last reworking (Ga) | | | | | | | | | |
| --- | --- | --- | --- | --- | --- | --- | --- | --- | --- | --- | --- | --- | --- | --- | --- | --- | --- | --- | --- |
|  |  |  | Best fit | Error + | | Error - | | Diameter range ^a^ | | Best fit | Error+ | | | Error- | | | Diameter Range ^a^ | | |
| XS1-S1 | Northern Branch | 22,610 | $3.70$ | | 0.04 | | 0.05 | | 2.0-4.0 km | 3.50 | | 0.02 | | | 0.02 | | | 0.5-1km | |
| XS1-S2 | Northern Branch | 8,324 | $3.60$ | | 0.05 | | 0.09 | | 1.0-3.5 km | $-$^b^ | | | | | | | | | |
| XS1-S3 | Northern Branch | 1,919 | 3.50 | | 0.07 | | 0.10 | | 0.7-1.4 km | $-$ | | | | | | | | | |
| XS2-S1 | Northern Branch | 7,389 | $-$ | | | | | | | 3.50 | | 0.05 | | | 0.09 | | | 1-1.4 km | |
| XS2-S2 | Northern Branch | 4,001 | $-$ | | | | | | | 3.50 | | 0.03 | | | 0.04 | | | 0.5-1.4 km | |
| XS2-S3 | Northern Branch | 2,124 | $-$ | | | | | | | 2.60 | | 0.40 | | | 0.50 | | | 0.5-1.4 km | |
| XS2-S4 | Northern Branch | 1,119 | $-$ | | | | | | | 0.89 | | 0.1 | | | 0.1 | | | 0.2-0.5km | |
| XS3-S1 | Northern Branch | 10,590 | 3.90 | | 0.03 | | 0.03 | | 2.0-4.0 km | $-$ | | | | | | | | | |
| XS3-S2 | Northern Branch | 7,128 | $-$ | | | | | | | $-$ | | | | | | | | | |
| XS4-S2 | Northern Branch | 2,243 | 3.50 | | 0.07 | | 0.10 | | 0.8-1.4 km | 1.30 | | | 0.08 | | | 0.08 | | | 0.2-0.5 km |
| XS5-S2 | Northern Branch | 15,120 | 3.50 | | 0.04 | | 0.05 | | 1.0-1.4 km | $-$ | | | | | | | | | |
| XS1-S1 | Southern Branch | 21,680 | 3.60 | | 0.03 | | 0.03 | | 1.0-2.0 km | 2.30 | | | 0.07 | | | 0.07 | | | 0.3-1.0 km |
| XS1-S2 | Southern Branch | 6,189 | $-$ | | | | | | | 2.00 | | | 0.20 | | | 0.20 | | | 0.5-1.4 km |
| XS1-S3 | Southern Branch | 3,168 | $-$ | | | | | | | $-$ | | | | | | | | | |
| XS2-S1 | Southern Branch | 15,720 | $-$ | | | | | | | 3.00 | | | 0.10 | | | 0.10 | | | 0.5-1.0 km |
| XS2-S2 | Southern Branch | 3,992 | 3.50 | | 0.07 | | 0.10 | | 0.9-1.4 km | $-$ | | | | | | | | | |
| XS2-S3 | Southern Branch | 2,932 | $-$ | | | | | | | 0.46 | | | 0.06 | | | 0.06 | | | 0.3-0.5 km |
| XS3-S2 | Southern Branch | 3,509 | 3.50 | | 0.09 | | 0.20 | | 1.0-2.0 km | 2.00 | | | 0.30 | | | 0.30 | | | 0.5-1.0 km |
| XS3-S3 | Southern Branch | 1,262 | $-$ | | | | | | | 0.69 | | | 0.06 | | | 0.06 | | | 0.1-0.3 km |
| XS4-S2 | Southern Branch | 8,533 | 3.50 | | 0.06 | | 0.10 | | 1.0-1.5 km | 2.40 | | | 0.20 | | | 0.20 | | | 0.5-1.0 km |
| NAS1 | Northern Branch | 904 | $-$ | | | | | | | 1.60 | | | 0.10 | | | 0.10 | | | 0.1-0.7 km |
| NAS2 | Northern Branch | 944 | $-$ | | | | | | | 1.20 | | | 0.20 | | | 0.20 | | | 0.3-0.7 km |
| NAS3 | Northern Branch | 8,285 | $-$ | | | | | | | 2.00 | | | 0.20 | | | 0.20 | | | 0.5-1.4 km |
| NAS4 | Northern Branch | 2,505 | 3.50 | | 0.07 | | 0.10 | | 0.8-1.4km | 1.80 | | | 0.10 | | | 0.10 | | | 0.3-0.6 km |
| NAS5 | Northern Branch | 5,065 | $-$ | | | | | | | 2.40 | | | 0.30 | | | 0.30 | | | 0.5-1.4 km |
| NAS6 | Northern Branch | 2,759 | $-$ | | | | | | | 2.30 | | | 0.40 | | | 0.40 | | | 0.5-1.0 km |
| NAS7 | Northern Branch | 6,490 | $-$ | | | | | | | 3.30 | | | 0.10 | | | 0.20 | | | 0.7-1.0 km |
| NAS8 | Northern Branch | 7,686 | $-$ | | | | | | | 3.20 | | | 0.10 | | | 0.20 | | | 0.7-1.4 km |
| SAS1 | Southern Branch | 6,226 | 3.30 | | 0.10 | | 0.50 | | 0.9-1.4 km | $-$ | | | | | | | | | |
| SAS2 | Southern Branch | 3,777 | $-$ | | | | | | | 2.10 | | | 0.30 | | | 0.30 | | | 0.5-1.0 km |
| SAS3 | Southern Branch | 1,011 | $-$ | | | | | | | 2.9 | | | 0.40 | | | 0.60 | | | 0.5-1.0 km |
| CS1 | Connection N-S | 701 | 3.20 | | 0.20 | | 0.70 | | 0.5-1.0 km | 1.10 | | | 0.01 | | | 0.01 | | | 0.1-0.3 km |
| CS2 | Connection N-S | 1,522 | $-$ | | | | | | | 2.10 | | | 0.50 | | | 0.50 | | | 0.5-1.4 km |
| CS3 | Connection N-S | 1,821 | 3.20 | | 0.01 | | 0.20 | | 0.3-0.5 km | $-$ | | | | | | | | | |

^a^ Crater diameter range used to determine the model age. Such ranges are comprised by randomly distributed diameters that fit well the production function

^b^ No model age determined due to either not having enough randomly distributed diameters that fit this part of the CSDF or insufficient crater population to infer about older reworking periods


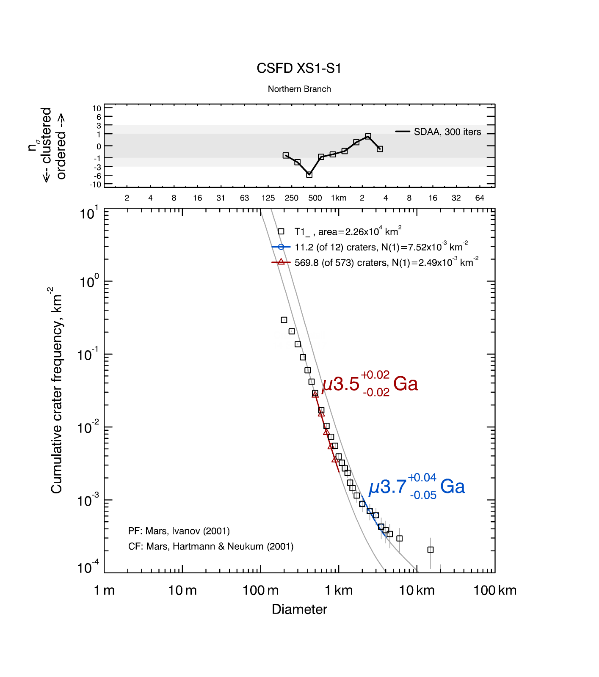

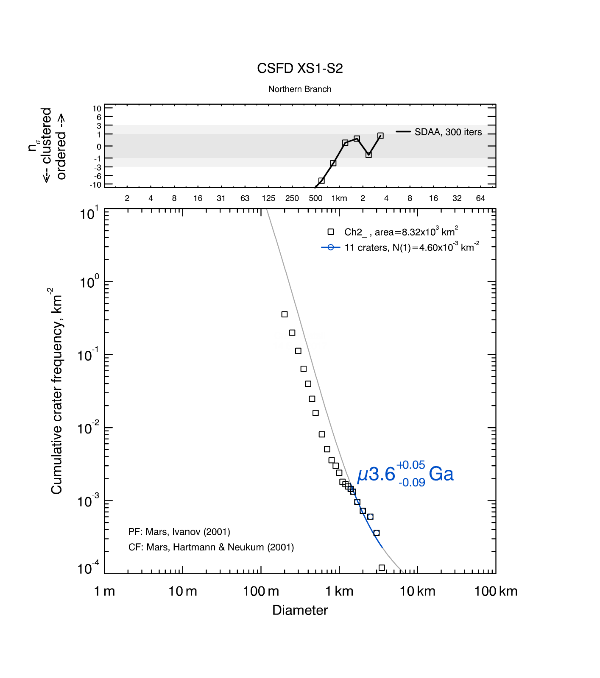

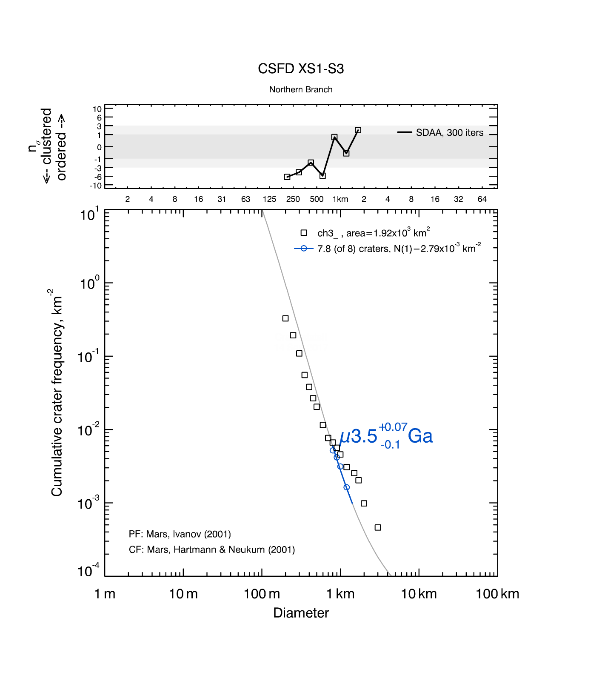


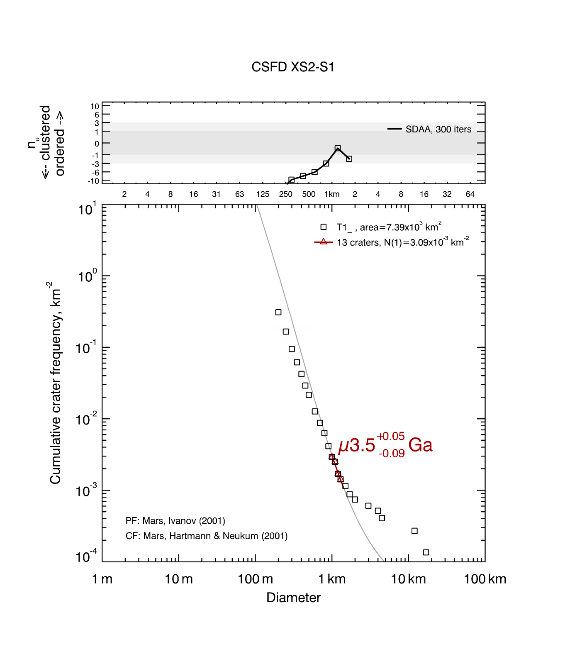

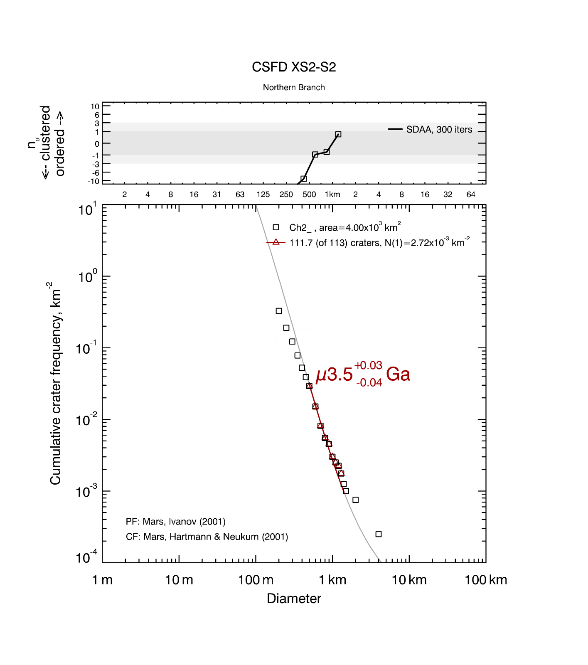

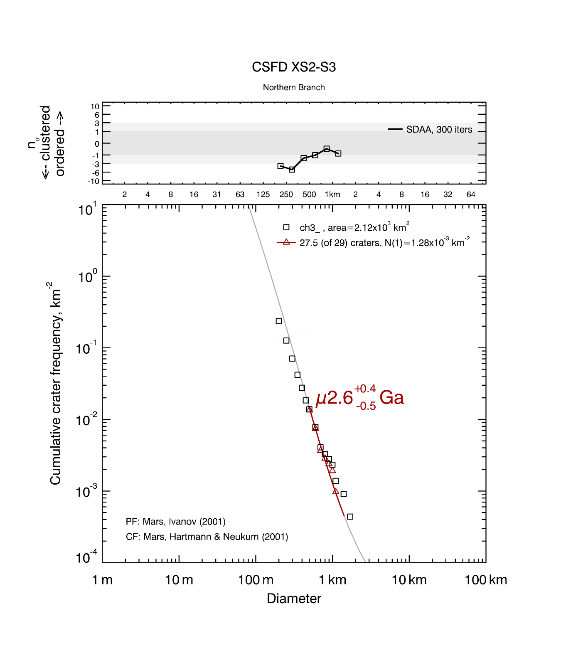


**Supp. Fig. 18. Crater size-frequency distributions displaying the formation time and/or last resurfacing of surfaces within the cross sections 1 & 2 of North Kasei.** The red fitting line represents the formation time of these surfaces, whereas the blue fitting line corresponds to the last reworking. A minimum of 4 bins (using a pseudo-log binning choice) were required to perform the model age estimate. These surfaces are located within Kasei Valles North.


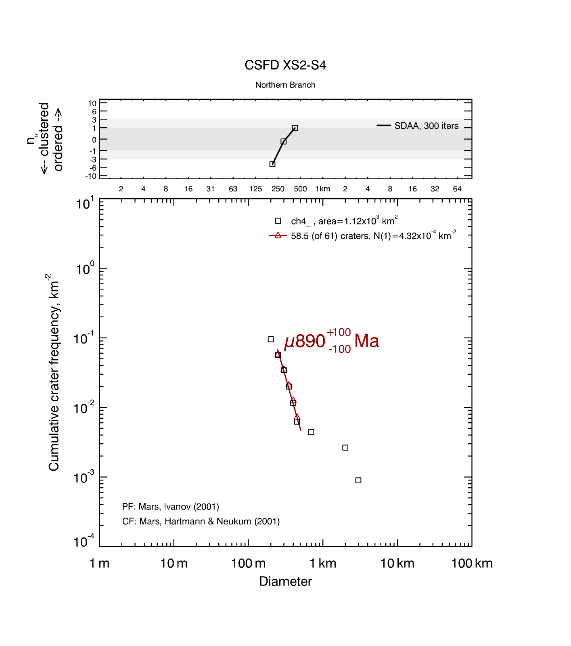

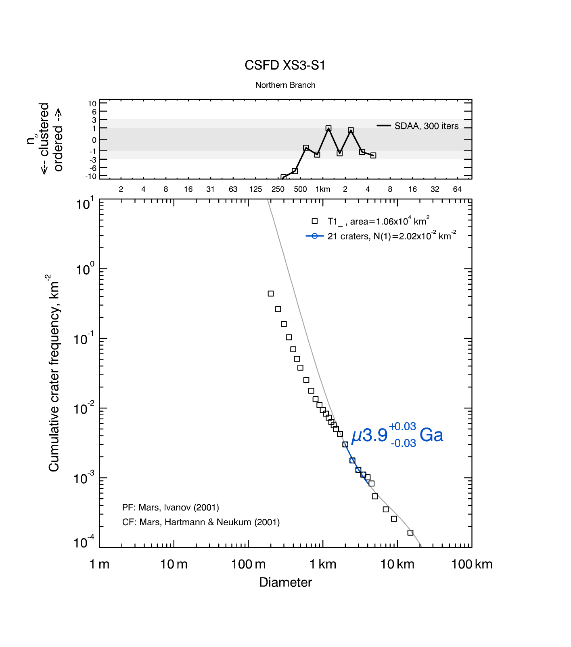

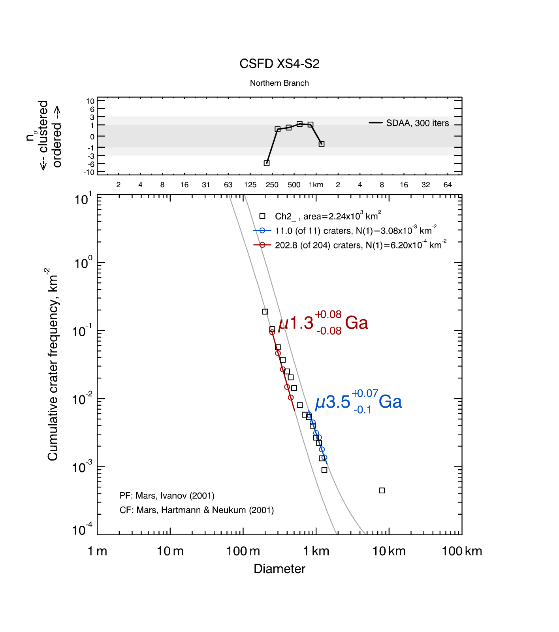


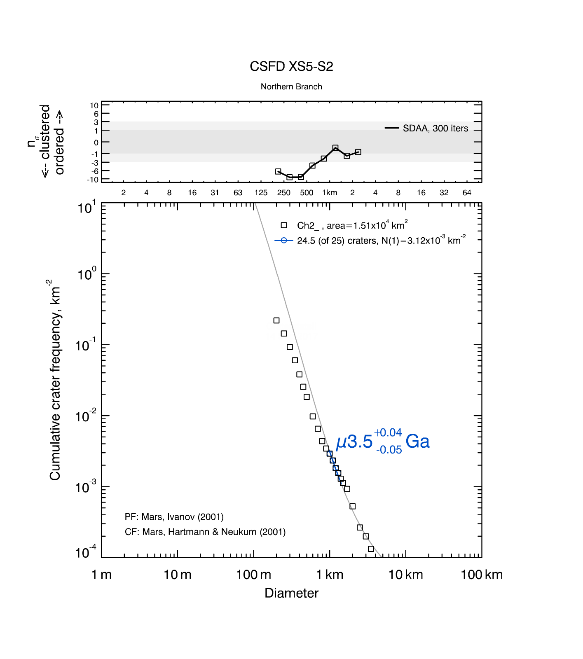

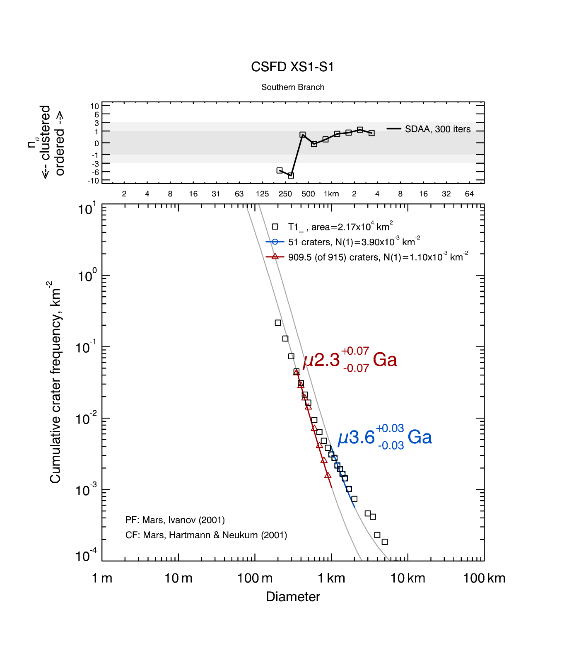

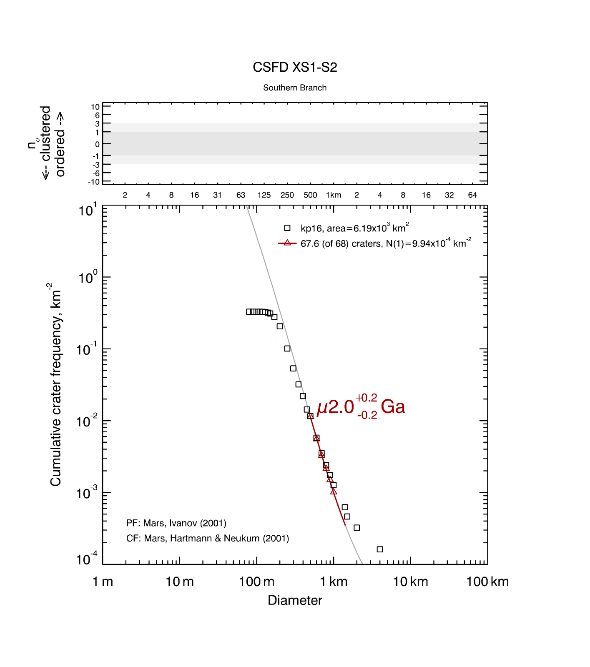


**Supp. Fig. 19. Crater size-frequency distributions displaying the formation time and/or last resurfacing of surfaces within the cross sections 2 , 3 & 4 of North Kasei and cross section 1 of South Kasei.** The red fitting line represents the formation time of these surfaces, whereas the blue fitting line corresponds to the last reworking. A minimum of 4 bins (using a pseudo-log binning choice) were required to perform the model age estimate. These surfaces are located within Kasei Valles North and South.


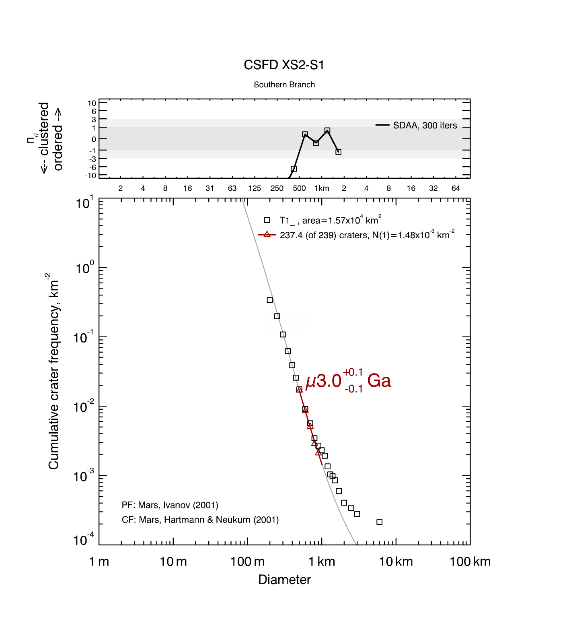

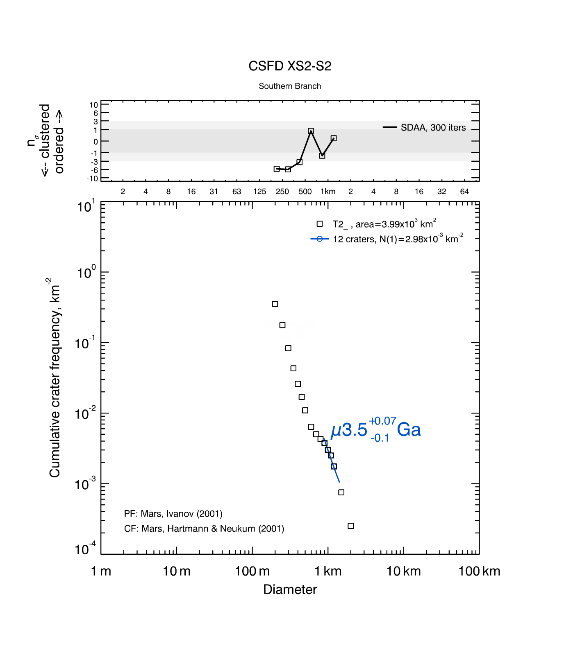

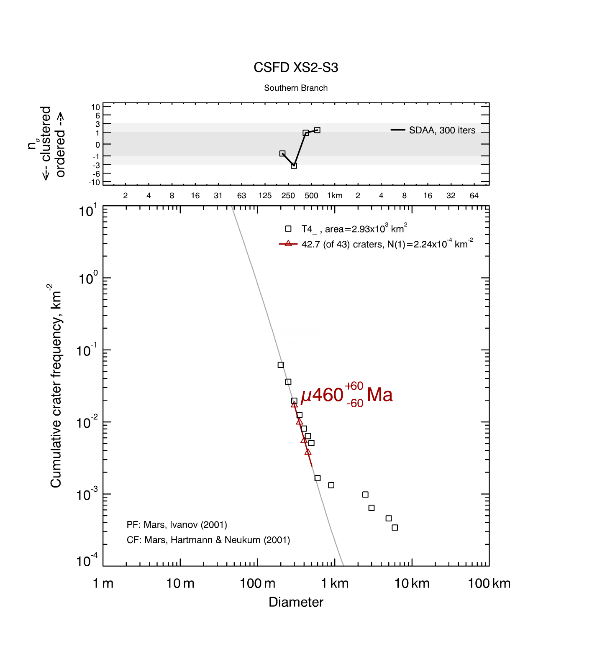


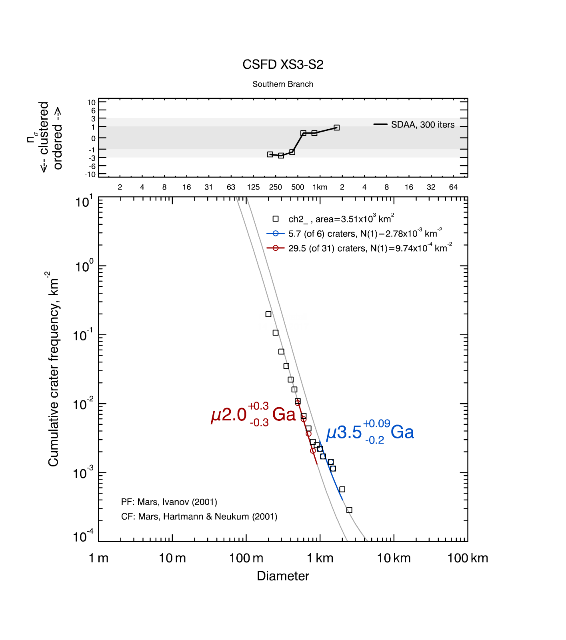

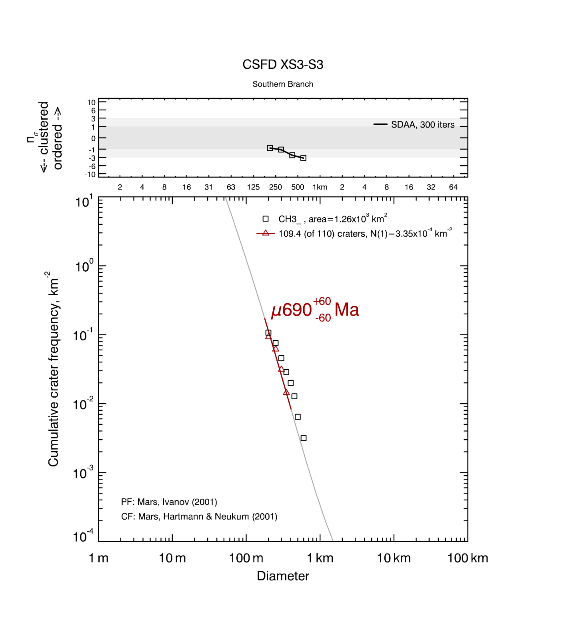

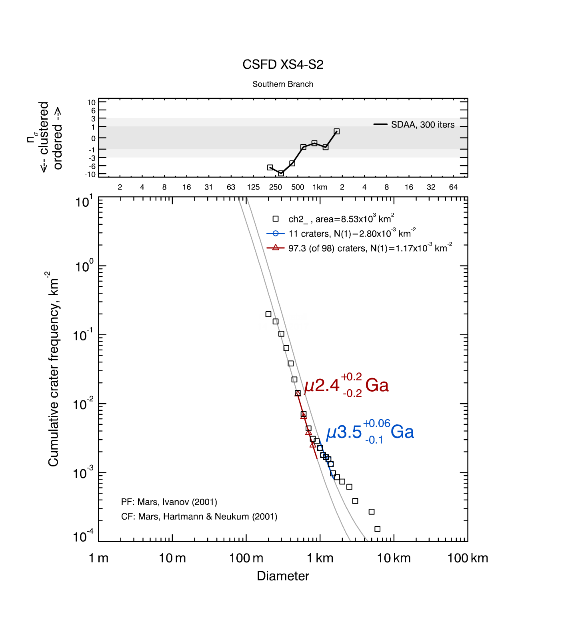


**Supp. Fig. 20. Crater size-frequency distributions displaying the formation time and/or last resurfacing of surfaces within the cross sections 1 , 2 , 3 & 4 of South Kasei.** The red fitting line represents the formation time of these surfaces, whereas the blue fitting line corresponds to the last reworking. A minimum of 4 bins (using a pseudo-log binning choice) were required to perform the model age estimate. These surfaces are located within Kasei Valles South.


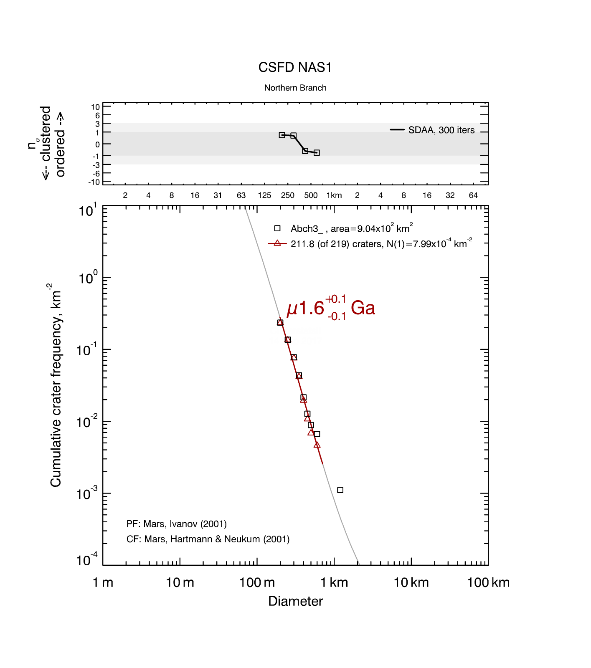

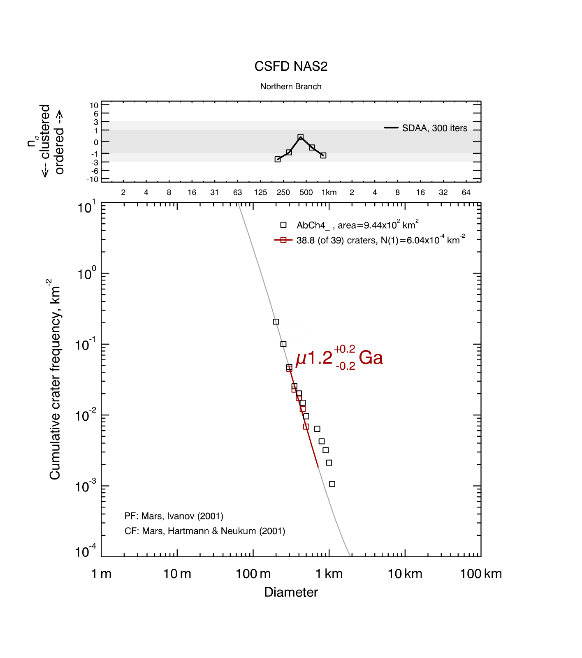

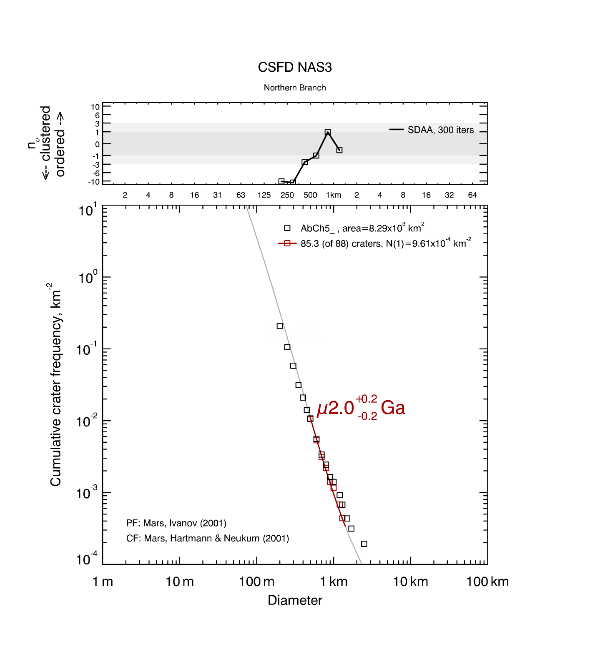


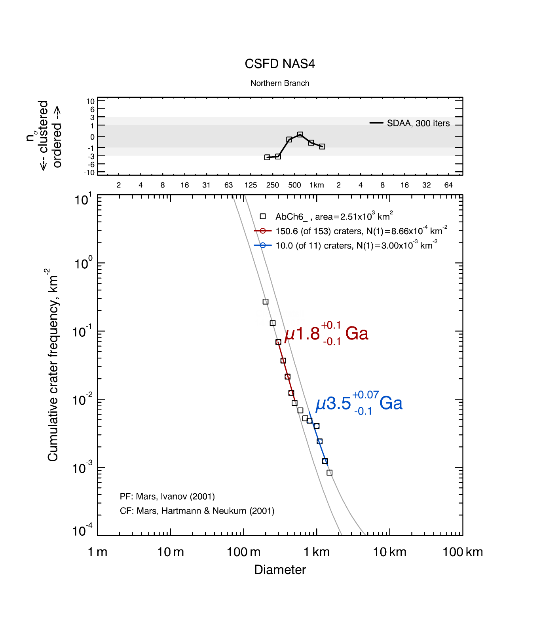

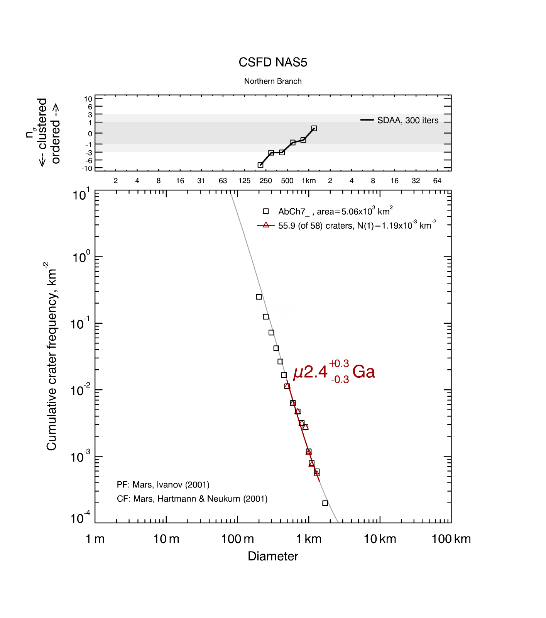

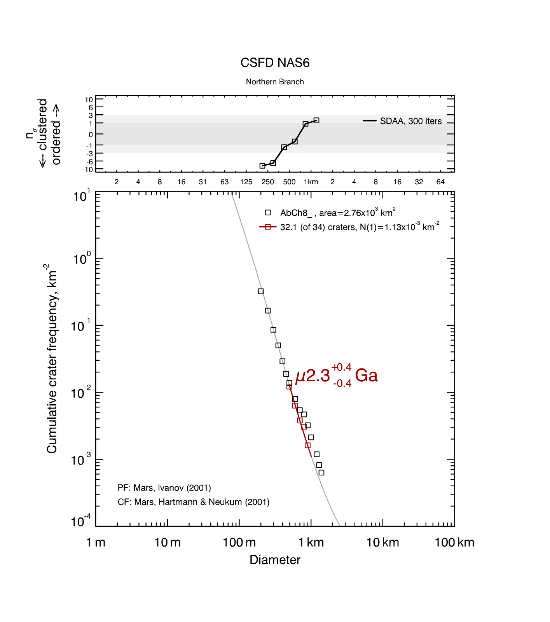


**Supp. Fig. 21. Crater size-frequency distributions displaying the formation time and/or last resurfacing of abandoned channels within North Kasei.** The red fitting line represents the formation time of these surfaces, whereas the blue fitting line corresponds to the last reworking. A minimum of 4 bins (using a pseudo-log binning choice) were required to perform the model age estimate. These surfaces are located within Kasei Valles North.


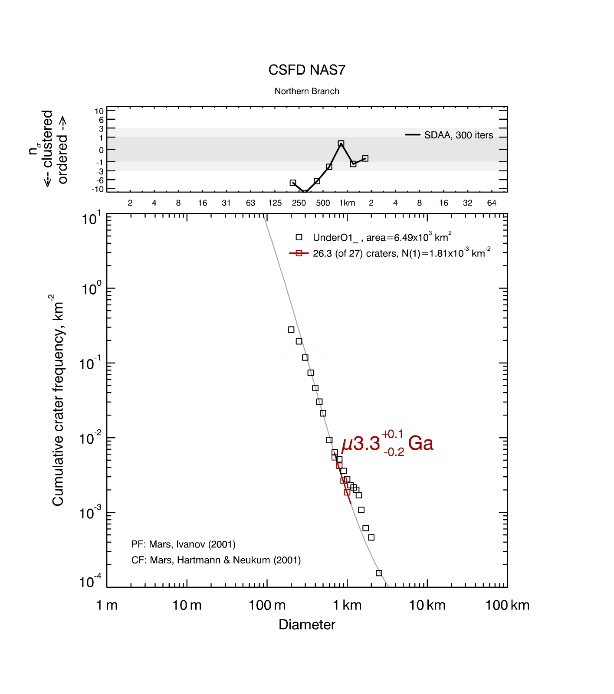

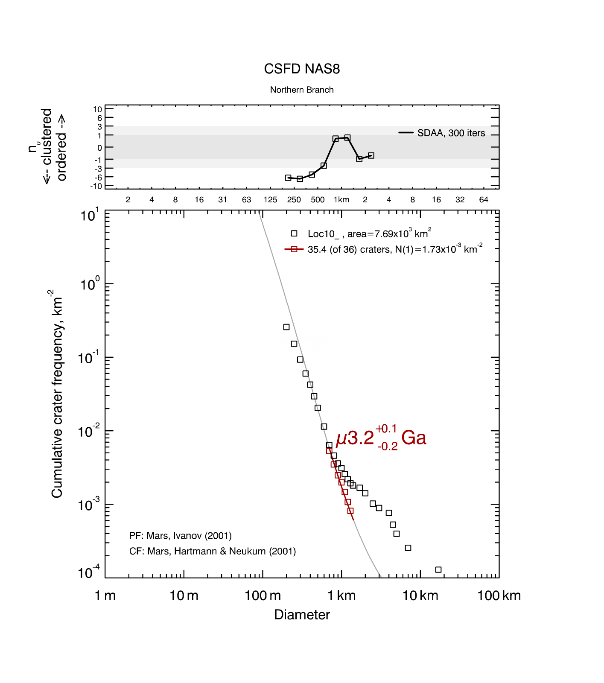

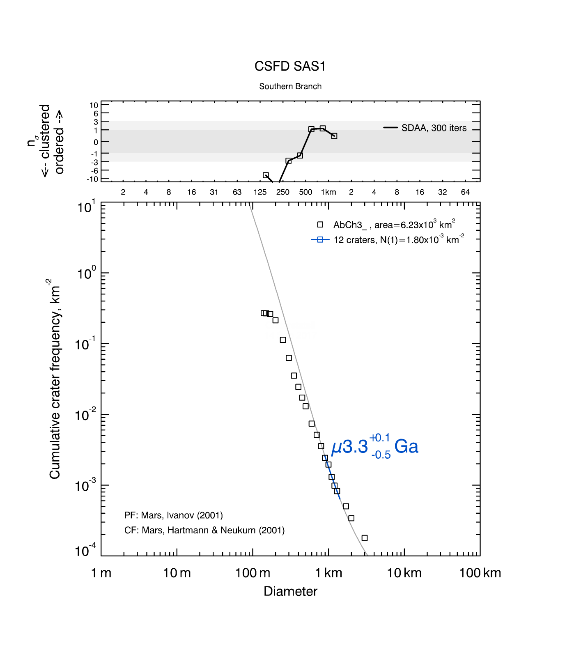

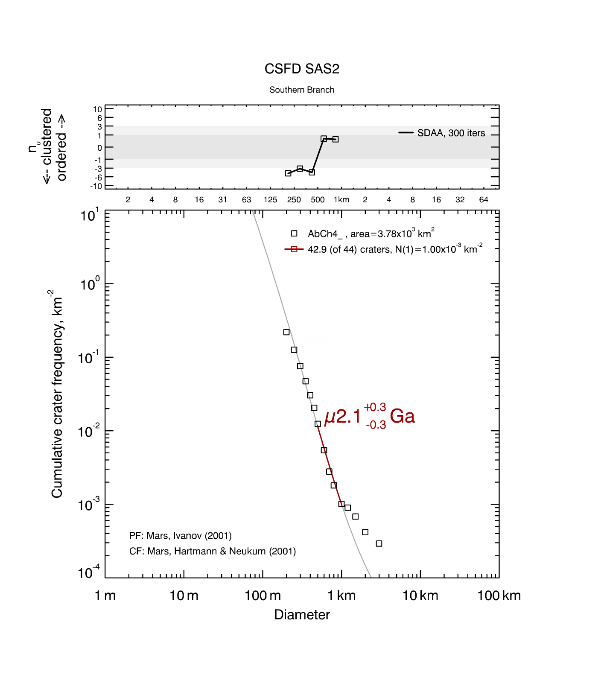

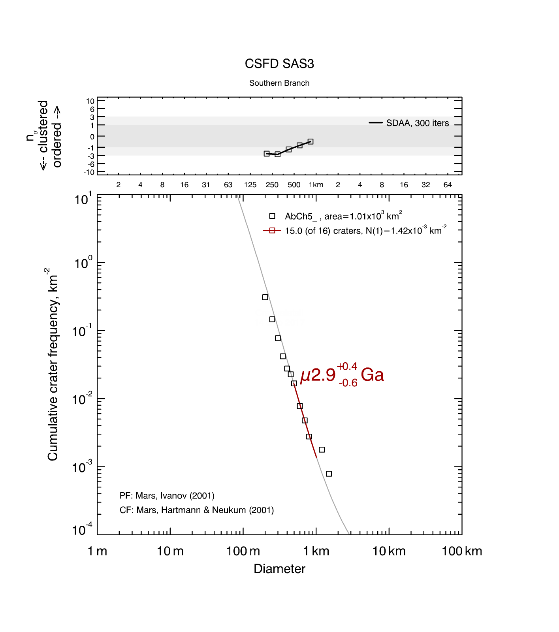

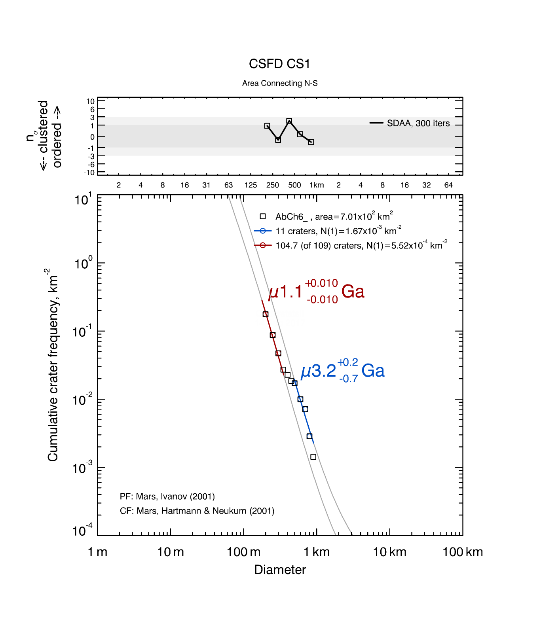


**Supp. Fig. 22. Crater size-frequency distributions displaying the formation time and/or last resurfacing of abandoned channels within North & South Kasei.** The red fitting line represents the formation time of these surfaces, whereas the blue fitting line corresponds to the last reworking. A minimum of 4 bins (using a pseudo-log binning choice) were required to perform the model age estimate. These surfaces are located within Kasei Valles North.


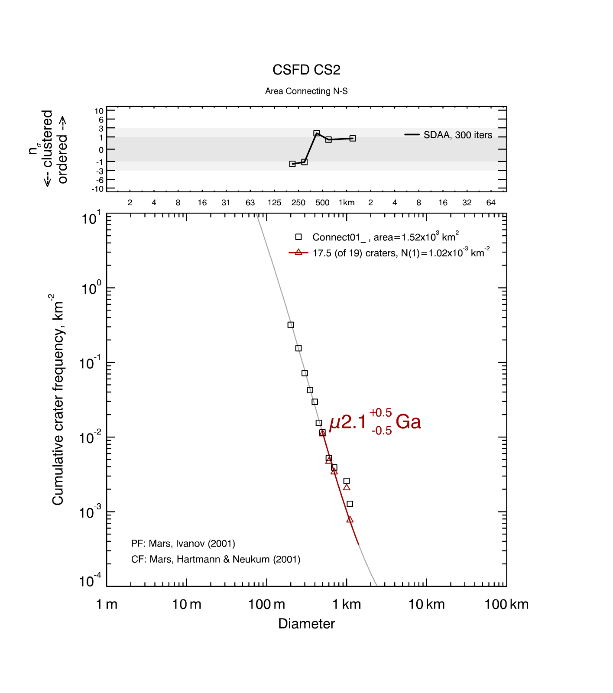

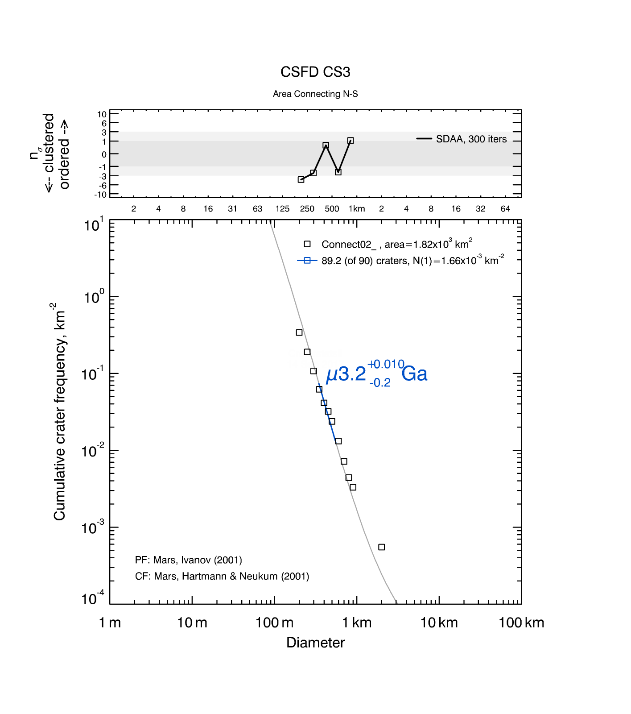


**Supp. Fig. 23. Crater size-frequency distributions displaying the formation time and/or last resurfacing of abandoned channels within North & South Kasei.** The red fitting line represents the formation time of these surfaces, whereas the blue fitting line corresponds to the last reworking. A minimum of 4 bins (using a pseudo-log binning choice) were required to perform the model age estimate. These surfaces are located within Kasei Valles North & South.
